# The GHP Scaling Limit of Uniform Spanning Trees in High Dimensions

**DOI:** 10.1007/s00220-023-04923-2

**Published:** 2024-03-04

**Authors:** Eleanor Archer, Asaf Nachmias, Matan Shalev

**Affiliations:** 1 Department of Mathematical Sciences, Tel Aviv University, 69978 Tel Aviv, Israel; 2 Équipe MODAL’X, Paris-Nanterre University, Nanterre, France

## Abstract

We show that the Brownian continuum random tree is the Gromov–Hausdorff–Prohorov scaling limit of the uniform spanning tree on high-dimensional graphs including the *d*-dimensional torus ${\mathbb {Z}}_n^d$ with $d>4$, the hypercube $\{0,1\}^n$, and transitive expander graphs. Several corollaries for associated quantities are then deduced: convergence in distribution of the rescaled diameter, height and simple random walk on these uniform spanning trees to their continuum analogues on the continuum random tree.

## Introduction

Consider the uniform spanning tree (UST) of the *d*-dimensional torus ${\mathbb {Z}}_n^d$ with $d>4$ or another transitive high-dimensional graph such as the hypercube $\{0,1\}^n$ or a transitive expander graph. In this paper we show that the Brownian continuum random tree (CRT), introduced by Aldous [1, 2], is the Gromov–Hausdorff–Prohorov (GHP) scaling limit of such USTs.

Convergence of such USTs to the CRT in the sense of finite dimensional distributions has been established in the work of Peres and Revelle [34]. The novelty of the current paper is proving that this convergence holds in the stronger GHP topology. This implies the convergence in distribution of some natural geometric quantities of the USTs (which were not known to converge prior to this work) and allows us to express their limiting distribution explicitly. For example, it follows from our work that the diameter and the height seen from a random vertex of these USTs, properly rescaled, converge to certain functionals of the Brownian excursion, as predicted by Aldous (see [2, Section 4]). Additionally, it implies that the simple random walk on these USTs converges to Brownian motion on the CRT. We discuss these implications in Sect. 1.3.

Our main result is as follows.

### Theorem 1.1

Let ${\mathcal {T}}_n$ be a uniformly drawn spanning tree of the *d*-dimensional torus ${\mathbb {Z}}_n^d$ with $d>4$. Denote by $d_{{\mathcal {T}}_n}$ the corresponding graph-distance on ${\mathcal {T}}_n$ and by $\mu _n$ the uniform probability measure on the vertices of ${\mathcal {T}}_n$. Then there exists a constant $\beta (d)>0$ such that

1 $$\begin{aligned} \left( {\mathcal {T}}_n,\frac{d_{{\mathcal {T}}_n}}{\beta (d) n^{d/2}},\mu _n\right) \overset{(d)}{\longrightarrow }\ ({\mathcal {T}},d_{{\mathcal {T}}},\mu ) \end{aligned}$$

where $({\mathcal {T}},d_{{\mathcal {T}}},\mu )$ is the CRT equipped with its canonical mass measure $\mu$ and $\overset{(d)}{\longrightarrow }$ means convergence in distribution with respect to the GHP distance between metric measure spaces.

### Remark 1.2

We take the convention of Aldous [2, Section 2] that the CRT is coded by two times standard Brownian excursion, although different normalizations are sometimes used elsewhere in the literature.

Our result shows that high-dimensional USTs exhibit a strong form of *universality*, a common phenomenon in statistical physics whereby above an *upper critical dimension*, the macroscopic behaviour of a system does not depend on the finer properties of the underlying network. For USTs the upper critical dimension is well-known to be four as for the closely related model of *loop-erased random walk* (LERW). Above dimension four LERW rescales to Brownian motion, see [24]. In lower dimensions the scaling limits are markedly different. On ${\mathbb {Z}}^2$ it was shown by Lawler, Schramm and Werner [25] that LERW rescales to $\textrm{SLE}_2$, and Barlow, Croydon and Kumagai [8] later established subsequential GHP scaling limits for the UST. This was later extended to full convergence in a result of Holden and Sun [18]. On ${\mathbb {Z}}^3$, much less is known, however the breakthrough works of Kozma [21] and Li and Shiraishi [28] on subsequential scaling limits of LERW enabled Angel, Croydon, Hernandez-Torres and Shiraishi [4] to show GHP convergence of the rescaled UST along a dyadic subsequence. Their scaling factors are given in terms of the LERW growth exponent in three dimensions, which was shown to exist by Shiraishi [37]. Finally, in four dimensions, a classical result of Lawler [23] computes the logarithmic correction to scaling under which the LERW on ${\mathbb {Z}}^4$ converges to Brownian motion. Schweinsberg [36] showed that with these logarithmic corrections to scaling, the finite-dimensional distributions of the UST on the four dimensional torus converge to those of the CRT, analogously to [34]. Various exponents governing the shape of the UST in ${\mathbb {Z}}^4$ are given in the recent works of Hutchcroft and Sousi [20] and Halberstam and Hutchcroft [17]. Our proof of GHP convergence does not encompass the four dimensional torus (see Problem 7.3).

In the rest of this section we first present the standard notation and definitions required to parse Theorem 1.1. We then state the most general version of our result, Theorem 1.6, handling other high-dimensional underlying graphs such as expanders and the hypercube. We close this section with a discussion of the various corollaries mentioned above and the organization of the paper.

### Standard notation and definitions

A **spanning tree** of a connected finite graph *G* is a connected subset of edges touching every vertex and containing no cycles. The **uniform spanning tree** (UST) is a uniformly drawn sample from this finite set. Given a tree *T* we denote by $d_T$ the graph distance metric on the vertices of *T*, i.e., $d_T(u,v)$ is the number of edges in the unique path between *u* and *v* in *T*.

We follow the setup of [31, Sections 1.3 and 6] and work in the space ${\mathbb {X}}_c$ of equivalence classes of (deterministic) metric measure spaces (mm-spaces) $(X,d,\mu )$ such that (*X*, *d*) is a compact metric space and $\mu$ is a Borel probability measure on (*X*, *d*), where we treat $(X,d,\mu )$ and $(X',d',\mu ')$ as equivalent if there exists a bijective isometry $\phi : X \rightarrow X'$ such that $\phi _* \mu = \mu '$ where $\phi _*\mu$ is the pushforward measure of $\mu$ under $\phi$. As is standard in the field, we will abuse notation and represent an equivalence class in ${\mathbb {X}}_c$ by a single element of that equivalence class.

We will now define the GHP metric on ${\mathbb {X}}_c$. First recall that if (*X*, *d*) is a metric space, the **Hausdorff distance** $d_H$ between two sets $A, A' \subset X$ is defined as

$$\begin{aligned} d_H(A, A') = \max \{ \sup _{a \in A} d(a, A'), \sup _{a' \in A'} d(a', A) \}. \end{aligned}$$

Furthermore, for $\varepsilon >0$ and $A\subset X$ we let $A^{\varepsilon } = \{ x \in X: d(x,A) < \varepsilon \}$ be the $\varepsilon$-fattening of *A* in *X*. If $\mu$ and $\nu$ are two measures on *X*, the **Prohorov distance** between $\mu$ and $\nu$ is given by

$$\begin{aligned}{} & {} d_P(\mu , \nu ) = \\{} & {} \quad \inf \{ \varepsilon > 0: \mu (A) \le \nu (A^{\varepsilon }) + \varepsilon \text { and } \nu (A) \le \mu (A^{\varepsilon }) + \varepsilon \text { for any closed set } A \subset X \}. \end{aligned}$$

#### Definition 1.3

Let $(X,d,\mu )$ and $(X',d',\mu ')$ be elements of ${\mathbb {X}}_c$. The **Gromov–Hausdorff–Prohorov** (GHP) distance between $(X,d,\mu )$ and $(X',d',\mu ')$ is defined as

$$\begin{aligned} d_{\textrm{GHP}}((X,d,\mu ),(X',d',\mu ')) = \inf \left\{ d_H (\phi (X), \phi '(X')) \vee d_P(\phi _* \mu , \phi _*' \mu ') \right\} , \end{aligned}$$

where the infimum is taken over all isometric embeddings $\phi : X \rightarrow F$, $\phi ': X' \rightarrow F$ into some common metric space *F*.

It is shown in [31, Theorem 6 and Proposition 8] that $({\mathbb {X}}_c, d_{\textrm{GHP}})$ is a Polish metric space. Denote by ${\mathcal {M}}_1({\mathbb {X}}_c^{\textrm{GHP}})$ the set of probability measures on $({\mathbb {X}}_c,d_{\textrm{GHP}})$ with the Borel $\sigma$-algebra. We say that a sequence of probability measures $\{{\mathbb {P}}_n\}_{n=1}^\infty \subset {\mathcal {M}}_1({\mathbb {X}}_c^{\textrm{GHP}})$ **converges weakly** to ${\mathbb {P}}\in {\mathcal {M}}_1({\mathbb {X}}_c^{\textrm{GHP}})$ if for any bounded continuous function $f:({\mathbb {X}}_c, d_{\textrm{GHP}}) \rightarrow {\mathbb {R}}$ we have $\lim _{n} {\mathbb {E}}_n f = {\mathbb {E}}f$, where ${\mathbb {E}}_n$ and ${\mathbb {E}}$ are the expectation operators corresponding to ${\mathbb {P}}_n$ and ${\mathbb {P}}$. As usual, if $\{X_n\}$ and *X* are random variables taking values in $({\mathbb {X}}_c, d_{\textrm{GHP}})$, we say that $X_n$ **converges in distribution** to *X*, written $X_n \overset{(d)}{\longrightarrow }\ X$, if the laws of $X_n$ converge weakly to that of *X*.

The CRT is a typical example of a random fractal tree and can be thought of as the scaling limit of critical (finite variance) Galton-Watson trees. As we shall explain in Sect. 3, we do not directly approach the CRT in this paper; therefore we have opted to omit the definition of the CRT and refer the reader to Le Gall’s comprehensive survey [26] for its construction (see also [2]) as a random element in ${\mathbb {X}}_c$. Except for this, by now we have stated all the necessary definitions required for Theorem 1.1.

### The general theorem

We now present the general version of Theorem 1.1 which will imply the GHP convergence of the UST on graphs like the hypercube $\{0,1\}^m$ or transitive expanders. Our assumptions on the underlying graph are stated in terms of random walk behavior but should be thought of as geometric assumptions. For a graph *G*, two vertices *x*, *y* and a non-negative integer *t* we write $p_t(x,y)$ for the probability that the lazy random walk starting at *x* will be at *y* at time *t*. When *G* is a finite connected regular graph on *n* vertices we define the **uniform mixing time** of *G* as

2 $$\begin{aligned} t_{\textrm{mix}}(G) = \min \left\{ t\ge 0: \max _{x,y\in G} \left| \frac{p_t(x,y)}{\pi (y)}-1\right| \le \frac{1}{2}\right\} , \end{aligned}$$

We will assume the following throughout the paper. This is the same assumption under which Peres and Revelle establish finite-dimensional convergence in [34].

#### Assumption 1.4

Let $\left\{ G_n \right\}$ be a sequence of finite connected vertex transitive graphs with $|G_n| = n$.

1. There exists $\theta < \infty$ such that $\displaystyle \sup _n \sum _{t=0}^{\sqrt{n}} (t+1) \sup _{x \in G_n} p_t(x,x) \le \theta$;
2. There exists $\alpha > 0$ such that $t_{\textrm{mix}}(G_n) = o (n^{\frac{1}{2} - \alpha })$ as $n \rightarrow \infty$.

Both items in Assumption 1.4 imply that the graph sequence is in some sense of dimension greater than four. The first item is a finite analogue of the condition that the expected number of intersections of two independent random walks is finite; in ${\mathbb {Z}}^d$ this happens if and only if $d>4$. The second item (which clearly holds on the torus on *n* vertices once $d>4$, since this has mixing time of order $n^{2/d}$) heuristically ensures that different parts of the ${{\,\textrm{UST}\,}}$ that are distance $\sqrt{n}$ apart behave asymptotically independently. We do not claim that these conditions are optimal (see the discussion in [30, Section 1.4]), but they are enough to yield convergence to the CRT in the most interesting cases.

As we will be able to prove some of our theorems in greater generality, we also introduce a slightly different set of assumptions.

#### Assumption 1.5

Let $\left\{ G_n \right\}$ be a sequence of finite connected graphs with $|G_n| = n$.

1. There exists $\theta < \infty$ such that $\displaystyle \sup _n \sum _{t=0}^{\sqrt{n}} (t+1) \sup _{x \in G_n} p_t(x,x) \le \theta$;
2. There exists $\alpha > 0$ such that $t_{\textrm{mix}}(G_n) = o (n^{\frac{1}{2} - \alpha })$ as $n \rightarrow \infty$.
3. There exists some $D\ge 1$ such that for all $n\in {\mathbb {N}}$ we have that $\frac{\max _{x\in G_n} \deg (x)}{\min _{y\in G_n} \deg (y)} \le D$ (we call such graphs balanced with parameter D).

The proof of GHP convergence requires two essential inputs: firstly the aforementioned result of Peres and Revelle [34], who proved a form of finite dimensional convergence, and secondly the “lower mass bound" condition, which is the subject of the present paper (we explain how these two inputs combine to give GHP convergence in Sect. 3 and Sect. 6). In view of future applications, such as the study of USTs of dense graphs carried out in [6], the lower mass bound of the present paper (Theorem 3.2) is proved under Assumption 1.5. However Theorem 1.6 below also requires transitivity since the result of [34] also assumes this.

#### Theorem 1.6

Let $\{ G_n \}$ be a sequence of graphs satisfying Assumption 1.4 and let ${\mathcal {T}}_n$ be a sample of ${{\,\textrm{UST}\,}}(G_n)$. Denote by $d_{{\mathcal {T}}_n}$ the graph distance on ${\mathcal {T}}_n$ and by $\mu _n$ the uniform probability measure on the vertices of ${\mathcal {T}}_n$. Then there exists a sequence $\{\beta _n\}$ satisfying $0<\inf _n \beta _n \le \sup _n \beta _n < \infty$ such that

$$\begin{aligned} \left( {\mathcal {T}}_n,\frac{d_{{\mathcal {T}}_n}}{\beta _n \sqrt{n}}, \mu _n \right) \overset{(d)}{\longrightarrow }\ \left( {\mathcal {T}}, d_{{\mathcal {T}}}, \mu \right) \end{aligned}$$

where $({\mathcal {T}},d_{{\mathcal {T}}},\mu )$ is the CRT equipped with its canonical mass measure $\mu$ and $\overset{(d)}{\longrightarrow }$ means convergence in distribution with respect to the GHP distance.

The sequence $\{\beta _n\}$ is inherited from the main result of Peres and Revelle, see [34, Theorem 1.2] (we restate this as Theorem 3.1 in this paper) and has a nice interpretation in terms of random walk intersection probabilities; we refer to [34, Lemma 8.1 and (17)] for the details. Note that Theorem 1.1 is not a special case of Theorem 1.6 since the latter does not guarantee a single scaling factor $\beta$, rather a sequence $\beta _n$ (which is the best one can hope for in the context of Theorem 1.6 since one can alternate between different graph sequences).

#### Proof of Theorem 1.1 given Theorem 1.6

For the torus ${\mathbb {Z}}_n^d$ with $d\ge 5$, Peres and Revelle proved that there exists $\beta (d)\in (0,\infty )$ such that [34, Theorem 1.2] holds with $\beta _n=\beta (d)$; see the choice of $\beta _n$ at the end of Section 3 of [34] as well as Lemma 8.1 and (17) in that paper. Hence, this and Theorem 1.6 readily imply Theorem 1.1. $\square$

Furthermore, see Lemma 1.3 and Section 9 of [34], in graphs where additionally two independent simple random walks typically avoid one another for long enough (see the precise condition in [34, Equation 6]), we can take $\beta _n \equiv 1$. This family of graphs includes the hypercube and transitive expanders with degrees tending to infinity. In the same spirit, for the *d*-dimensional torus, $\beta (d) \rightarrow 1$ as $d\rightarrow \infty$. Moreover, it is also immediate to see that Assumption 1.4 holds for a sequence of bounded degree transitive expanders (see for instance [34, Section 9]) and hence Theorem 1.6 holds for them as well.

### Corollaries

#### Pointed convergence

In order to establish some of the corollaries alluded to above, it will be useful to rephrase Theorem 1.6 in terms of *pointed convergence*. Roughly speaking, this means that we consider our spaces to be rooted, and we add a term corresponding to the distance between the roots in the embedding in Definition 1.3. We refer to [11, Section 2.2] for the precise definition, and in what follows we let ${\mathbb {X}}_c^{\text {pointed}}$ denote the space of compact rooted measure-metric spaces endowed with the pointed GHP topology as defined there.

We start with the following observation, which is a trivial consequence of a coupling characterization of the Prohorov distance (see [31, Proof of Proposition 6]).

##### Lemma 1.7

Given $(X, d, \mu ) \in {\mathbb {X}}_c$, let ${\mathcal {L}}((X, d, \mu , U))$ denote the law of $(X, d, \mu , U)$ in the space ${\mathbb {X}}_c^{\text {pointed}}$ where *U* is chosen according to $\mu$. Let *f* denote the mapping defined by $f((X, d, \mu )) = {\mathcal {L}}((X, d, \mu , U))$. Then *f* is continuous.

Due to transitivity, in our setting the root can be an arbitrary vertex $O_n$ rather than uniformly chosen. Combining Theorem 1.6 with Lemma 1.7 we deduce the following.

##### Theorem 1.8

(Pointed convergence). Let $\{ G_n \}$ be a sequence of graphs satisfying Assumption 1.4, let ${\mathcal {T}}_n$ be a sample of ${{\,\textrm{UST}\,}}(G_n)$ and let $O_n$ be an arbitrary vertex of $G_n$. Denote by $d_{{\mathcal {T}}_n}$ the graph distance on ${\mathcal {T}}_n$ and by $\mu _n$ the uniform probability measure on the vertices of ${\mathcal {T}}_n$. Then there exists a sequence $\{\beta _n\}$ satisfying $0<\inf _n \beta _n \le \sup _n \beta _n < \infty$ such that

$$\begin{aligned} \left( {\mathcal {T}}_n,\frac{d_{{\mathcal {T}}_n}}{\beta _n \sqrt{n}}, \mu _n, O_n \right) \overset{(d)}{\longrightarrow }\ \left( {\mathcal {T}}, d_{{\mathcal {T}}}, \mu , O \right) \end{aligned}$$

where $({\mathcal {T}},d_{{\mathcal {T}}},\mu , O)$ is the CRT equipped with its canonical mass measure $\mu$ and root *O*, and $\overset{(d)}{\longrightarrow }$ means convergence in distribution in ${\mathbb {X}}_c^{\text {pointed}}$.

#### Diameter distribution

The **diameter** of a metric space (*X*, *d*) is $\sup _{x,y\in X}d(x,y)$ and denoted by ${{\,\textrm{Diam}\,}}(X)$. When *X* is a tree, it is just the length of the longest path. The study of the diameter of random trees has an interesting history. Szekeres [38] proved in 1982 that the diameter $D_n$ of a uniformly drawn labeled tree on *n* vertices normalized by $n^{-1/2}$ converges in distribution to a random variable *D* with the rather unpleasant density

3 $$\begin{aligned}{} & {} f_D(y) = {\sqrt{2\pi }\over 3} \sum _{n\ge 1} e^{-b_{n,y}} \nonumber \\{} & {} \quad \Big ( {64 \over y^2} ( 4b_{n,y}^4 - 36b_{n,y}^3 +75 b_{n,y}^2 -30b_{n,y}) + {16 \over y^2} (2b_{n,y}^3 - 5b_{n,y}^2) \Big ) \,, \end{aligned}$$

where $b_{n,y} = 8(\pi n/y)^2$ and $y\in (0,\infty )$. Aldous [1, 2] showed that this tree, viewed as a random metric space, converges to the CRT and deduced that *D* is distributed as

4 $$\begin{aligned} 2 \cdot \sup _{0 \le t_1 < t_2 \le 1} \big ( e_{t_1} + e_{t_2} - 2 \inf _{t_1 \le t \le t_2} e_t \big )\,, \end{aligned}$$

where $\{e_t\}_{t \in [0,1]}$ is standard Brownian excursion. Curiously enough, up until 2015 the only known way to show that (4) has density (3) was to go via random trees and combine the Aldous and Szekeres results. Wang [39], prompted by a question of Aldous, gave a direct proof of this fact in 2015.

A uniformly drawn labeled tree on *n* vertices is just ${{\,\textrm{UST}\,}}(K_n)$ where $K_n$ is the complete graph on *n* vertices. Applying Theorem 1.6 we are able to extend Szekeres’ 1983 result to USTs of any sequence of graphs satisfying Assumption 1.4.

##### Corollary 1.9

Let $\{G_n\}$ be a sequence of graphs satisfying Assumption 1.4, let ${\mathcal {T}}_n$ be a sample of ${{\,\textrm{UST}\,}}(G_n)$ and let $\{\beta _n\}$ be the sequence guaranteed to exist by Theorem 1.6. Then

$$\begin{aligned} {{{\,\textrm{Diam}\,}}({\mathcal {T}}_n) \over \beta _n n^{1/2}} \overset{(d)}{\longrightarrow }\ D \,, \end{aligned}$$

where *D* is the diameter of the CRT, i.e., a random variable defined by either (3) or (4).

##### Proof

Let $D_n = {{\,\textrm{Diam}\,}}({\mathcal {T}}_n)$ and let $g:[0,\infty )\rightarrow {\mathbb {R}}$ be bounded and continuous. The function $h:{\mathbb {X}}_c\rightarrow {\mathbb {R}}$ defined by $h((X,d,\mu ))={{\,\textrm{Diam}\,}}(X)$ is continuous with respect to the GHP topology; indeed, for any two metric spaces $X_1$ and $X_2$ we have $|{{\,\textrm{Diam}\,}}(X_1) - {{\,\textrm{Diam}\,}}(X_2)|\le 2d_{\textrm{GHP}}(X_1,X_2)$. Thus the composition $g \circ h: {\mathbb {X}}_c \rightarrow {\mathbb {R}}$ is bounded and continuous. By Theorem 1.6 we conclude ${\mathbb {E}}\big [ g\circ h (({\mathcal {T}}_n, {d_{{\mathcal {T}}} \over \beta _n \sqrt{n}},\mu _n) \big ] \rightarrow {\mathbb {E}}[g\circ h (({\mathcal {T}},d,\mu ))]$ where $({\mathcal {T}},d,\mu )$ is the CRT. Therefore, ${\mathbb {E}}[g(D_n)] \rightarrow {\mathbb {E}}[g(D)]$ as required. $\square$

#### Height distribution

Given a rooted tree (*T*, *v*), the **height** of (*T*, *v*) is $\sup _{x\in T} d(v,x)$, i.e. the length of the longest simple path in *T* starting from *v*, and denoted by ${{\,\textrm{Height}\,}}(T,v)$. The study of the height of random trees predates the study of the diameter. In 1967, Rényi and Szekeres [35] found the limiting distribution of the height of a uniformly drawn labeled rooted tree on *n* vertices normalized by $n^{-1/2}$; we omit the precise formula this time (it is also unpleasant). Aldous [1, 2] realized that the limiting distribution is that of the maximum of the Brownian excursion.

The following corollary is an immediate consequence of Theorem 1.8. The proof goes along the same lines as the proof of Corollary 1.9; we omit the details.

##### Corollary 1.10

Let $\{G_n\}$ be a sequence of graphs satisfying Assumption 1.4, let ${\mathcal {T}}_n$ be a sample of ${{\,\textrm{UST}\,}}(G_n)$ and let $\beta _n$ be the sequence guaranteed to exist by Theorem 1.6. Let $v_n$ be an arbitrary vertex of $G_n$. Then

$$\begin{aligned} {{{\,\textrm{Height}\,}}({\mathcal {T}}_n,v_n) \over \beta _n n^{1/2}} \overset{(d)}{\longrightarrow }\ 2 \sup _{t\in [0,1]} e_t \,, \end{aligned}$$

where $\{e_t\}_{t\in [0,1]}$ is standard Brownian excursion.

#### SRW on the UST converges to BM on the CRT

A particularly nice application of Theorem 1.6 together with [11, Theorem 1.2] allows us to deduce that the simple random walk (SRW) on ${{\,\textrm{UST}\,}}(G_n)$ rescales to Brownian motion on the CRT. The latter object was first defined by Aldous in [2, Section 5.2] and formally constructed by Krebs [22]. In what follows, we let $P^{(O)}( \cdot )$ denote the law of Brownian motion on the CRT as constructed by Krebs, started from *O*.

In particular, the law of a random walk on ${{\,\textrm{UST}\,}}(G_n)$ is characterized by two associated quantities known as the resistance metric and speed measure on ${{\,\textrm{UST}\,}}(G_n)$. The result of [11, Theorem 1.2] shows that if a sequence of graphs equipped with a resistance metric and speed measure converge in the GHP topology, then the laws of the associated random walks also converge. In the case of a simple random walk on ${{\,\textrm{UST}\,}}(G_n)$, the relevant resistance metric coincides with the graph metric, and the speed measure is equal to the degree measure, which, as we show below, is close to the uniform measure in the GHP topology. We refer to [14] for more background on the connection between stochastic processes, resistance metrics and speed measures.

Also let $(X_n(m))_{m \ge 0}$ be a simple random walk on ${\mathcal {T}}_n$ and let $P^{(O_n)}_n$ denote the law of the rescaled process $\left( \frac{1}{\beta _n \sqrt{n}} X_n(2\beta _n n^{\frac{3}{2}}t)\right) _{t \ge 0}$ started from $O_n$.

##### Theorem 1.11

Let $\{ G_n \}$ be a sequence of graphs satisfying Assumption 1.4, let ${\mathcal {T}}_n$ be a sample of ${{\,\textrm{UST}\,}}(G_n)$, and let $(X_n(m))_{m \ge 0}$ be a simple random walk on ${\mathcal {T}}_n$. Then there exists a probability space $\Omega$ on which the convergence of Theorem 1.8 holds almost surely, and furthermore, on this probability space, for almost every $\omega \in \Omega$ the spaces $(({\mathcal {T}}_n, d_n, \mu _n, O_n))_{n \ge 1}$ and $({\mathcal {T}}, d, \mu , O)$ can be embedded into a common metric space $(X', d')(\omega )$ so that

5 $$\begin{aligned} P^{(O_n)}_n \left( \left( \frac{1}{\beta _n \sqrt{n}} X_n(2\beta _n n^{\frac{3}{2}}t)\right) _{t \ge 0} \in \cdot \right) \rightarrow P^{(O)}\left( (B_t)_{t \ge 0} \in \cdot \right) \end{aligned}$$

weakly as probability measures on the space $D({\mathbb {R}}^{\ge 0}, X'(\omega ))$ of càdlàg functions equipped with the topology of uniform convergence on compact time intervals.

##### Proof

The existence of such a probability space $\Omega$ follows from the Skorohod representation theorem since the space of pointed compact mm-spaces endowed with a finite measure is separable by [31, Theorem 6 and Proposition 8]. The theorem now follows from [11, Theorem 1.2] and two additional observations.

Firstly, if $\nu _n (x) = \deg x$, then $d_{\textrm{GHP}}\left( ({\mathcal {T}}_n, \frac{d_{{\mathcal {T}}_n}}{\beta _n \sqrt{n}}, \mu _n), ({\mathcal {T}}_n, \frac{d_{{\mathcal {T}}_n}}{\beta _n \sqrt{n}}, \frac{1}{2n}\nu _n) \right) \le \frac{1}{\beta _n \sqrt{n}}$, so that

$$\begin{aligned} \left( {\mathcal {T}}_n,\frac{d_{{\mathcal {T}}_n}}{\beta _n \sqrt{n}}, \frac{1}{2n}\nu _n \right) \overset{(d)}{\longrightarrow }\ \left( {\mathcal {T}}, d, \mu \right) \end{aligned}$$

with respect to the $\textrm{GHP}$ distance as a consequence of Theorem 1.6 and the triangle inequality. It therefore follows from [11, Theorem 1.2] that if $(Y_n(t))_{t \ge 0}$ is a continuous time SRW on $G_n$ with an $\textsf {exp}(1)$ holding time at each vertex, then

6 $$\begin{aligned} \left( \frac{1}{\beta _n \sqrt{n}} Y_n(2\beta _n n^{\frac{3}{2}}t)\right) _{t \ge 0} \overset{(d)}{\longrightarrow } (B_t)_{t \ge 0} \end{aligned}$$

as $n \rightarrow \infty$, almost surely on $\Omega$. This result then transfers to the SRW sequence $(X_n(\cdot ))_{n \ge 1}$ in place of $(Y_n(\cdot ))_{n \ge 1}$ by standard arguments using the strong law of large numbers and continuity of the limit process. We refer to [5, Section 4.2] for an example of such an argument. $\square$

We can similarly obtain joint convergence of rescaled mixing times [13] and transition densities [12]. In addition, it was recently verified by Noda that local times also converge [32]. The latter requires a slightly stronger input than Theorem 1.8, however the required condition is straightforward consequence of our lower mass bound, as was obtained by Noda in the recent paper [32, Section 8.2].

### Organization

We begin with some preliminaries in Sect. 2 where we introduce the standard definitions of loop-erased random walk, mixing time and capacity which are central to the proof. We also record some stochastic domination properties of USTs, and prove there a general result regarding negative correlations of certain expected volumes in the ${{\,\textrm{UST}\,}}$ (see Claim 2.12).

Next in Sect. 3 we present the main argument of the proof, while delegating two useful estimates, Theorem 3.3 and Theorem 3.6, to Sect. 4, and a third useful estimate, Lemma 3.7, to Sect. 5. In Sect. 6 we present a necessary though rather straightforward abstract argument combining the result of Sect. 3 with the results of [34] to yield Theorem 1.6. Lastly, in Sect. 7 we present some concluding remarks and open questions.

A note on constants: throughout the proof we take limits with respect to three parameters $\varepsilon , c$ and *n*. In order to keep track of the precise dependence of different constants on each of these parameters, we have chosen to keep constants explicit rather than using big-O notation. In most proofs the precise value of constants will not be important.

## Preliminaries

In this section we provide an overview of the tools used to prove Theorem 1.6. Throughout the section, we assume that $G=(V,E)$ is a finite connected graph with *n* vertices. We will use the following conventions.

- For an integer $m\ge 1$ we write $[m]=\{1,\ldots , m\}$.
- For two positive sequences *t*(*n*), *r*(*n*) we write $t \sim r$ when $t(n)/r(n) \rightarrow 1$.
- For two positive sequences *t*(*n*), *r*(*n*) we write $t \gg r$ when $t(n)/r(n) \rightarrow \infty$.
- For ease of reading, we omit floor and ceiling signs (all of the relevant quantities are large).
- Through the rest of this paper, the *random walk* on a graph equipped with positive edge weights is the random walk that stays put with with probability 1/2 and otherwise jumps to a random neighbor with probability proportional to the weight of the corresponding edge. If no edge weights are specified, then they are all unit weights.

### Loop-erased random walk and Wilson’s algorithm

Wilson’s algorithm [40], which we now describe, is a widely used algorithm for sampling ${{\,\textrm{UST}\,}}$s. A **walk** $X=(X_0, \ldots X_L)$ of length $L\in {\mathbb {N}}$ is a sequence of vertices where $(X_i, X_{i+1})\in E(G)$ for every $0 \le i \le L-1$. For an interval $J=[a,b]\subset [0,L]$ where *a*, *b* are integers, we write *X*[*J*] for $\{X_i\}_{i=a}^{b}$. Given a walk, we define its **loop erasure** $Y = {{\,\textrm{LE}\,}}(X) = {{\,\textrm{LE}\,}}(X[0,L])$ inductively as follows. We set $Y_0 = X_0$ and let $\lambda _0 = 0$. Then, for every $i\ge 1$, we set $\lambda _i = 1+\max \{t \mid X_t = Y_{\lambda _{i-1}}\}$. If $\lambda _i \le L$ we set $Y_i = X_{\lambda _i}$ and otherwise, we halt the process and set ${{\,\textrm{LE}\,}}(X)=\{Y_k\}_ {k=0}^{i-1}$. The times $\left\{ \lambda _k(X)\right\} _{k=0}^{|{{\,\textrm{LE}\,}}(X)| - 1}$ are the **times contributing to the loop-erasure** of the walk *X*. When *X* is a random walk starting at some vertex $v\in G$ and terminated when hitting a set of vertices *W* (*L* is now random), we say that ${{\,\textrm{LE}\,}}(X)$ is the **loop erased random walk** (${{\,\textrm{LERW}\,}}$) from *v* to *W*. Note that ${{\,\textrm{LE}\,}}(X)$ is obtained by erasing the loops from *X* in chronological order as they appear.

To sample a ${{\,\textrm{UST}\,}}$ of a finite connected graph *G* we begin by fixing an ordering of the vertices of $V=(v_1,\ldots , v_n)$. At the first step, let $T_1$ be the tree containing $v_1$ and no edges. At each step $i>1$, sample a ${{\,\textrm{LERW}\,}}$ from $v_i$ to $T_{i-1}$ and set $T_i$ to be the union of $T_{i-1}$ and the ${{\,\textrm{LERW}\,}}$ that has just been sampled. We terminate this algorithm with $T_n$. Wilson [40] proved that $T_n$ is distributed as ${{\,\textrm{UST}\,}}(G)$. An immediate consequence is that the path between any two vertices in ${{\,\textrm{UST}\,}}(G)$ is distributed as a ${{\,\textrm{LERW}\,}}$ between those two vertices. This was first shown by Pemantle [33].

To understand the lengths of loops erased in ${{\,\textrm{LERW}\,}}$ we will need the notion of the **bubble sum**. Let *G* be a graph and let *W* be a non empty subset of vertices of *G*. For every two vertices $u,w\in V(G)$, define

$$\begin{aligned} \textbf{p}_{W}^t(u,w) = {\mathbb {P}}_u(X_t = w, X[0,t]\cap W = \emptyset ) \,, \end{aligned}$$

where *X* is a random walk on *G*. We define the *W*-bubble sum by

$$\begin{aligned} {\mathcal {B}}_W(G):= \sum _{t=0}^{\infty }(t+1)\sup _{v\in V} \textbf{p}_{W}^t(v,v). \end{aligned}$$

Note that since the random walk on *G* is an irreducible Markov chain on a finite state space, we have that ${\mathbb {P}}(X[0,t] \cap W = \emptyset )$ decays exponentially in *t* and hence this sum is always finite. Another bubble-sum we will consider is when the random walk is killed at a geometric time (rather than when hitting a set *W*). Let $T_\zeta$ be an independent geometric random variable with mean $\zeta >1$. We define

$$\begin{aligned} \textbf{p}_{\zeta }^t (u,w) = {\mathbb {P}}_u(X_t = w, T_\zeta >t), \quad {\mathcal {B}}_\zeta (G):= \sum _{t=0}^{\infty }(t+1)\sup _{v\in V} \textbf{p}_{\zeta }^t(v,v). \end{aligned}$$

#### Definition 2.1

We say that a random walk *X* on a finite connected graph *G* starting from an arbitrary vertex is **bubble-terminated** with bubble-sum bounded by $\psi$ if it is killed upon hitting some set *W* and $B_W(G) \le \psi$, or alternatively, if it is killed at time $T_\zeta -1$ and $B_{\zeta }(G) \le \psi$.

Both bubble-sums allow us to bound the size of the loops erased in the loop-erasure process. As in [19] and [30, Claim 3.2] we have the following.

#### Claim 2.2

Let *G* be a finite connected graph and *X* be a bubble-terminated random walk on *G* with bubble-sum bounded by $\psi$. For any finite simple path $\gamma$ such that ${\mathbb {P}}({{\,\textrm{LE}\,}}(X)=\gamma )>0$ of length *L* we have that the random variables

$$\begin{aligned} \left\{ \lambda _{i+1}(X) - \lambda _i(X) \right\} _{i=0}^{L-1} \end{aligned}$$

are independent conditionally on $\{{{\,\textrm{LE}\,}}(X) = \gamma \}$ and furthermore

$$\begin{aligned} {\mathbb {E}}[\lambda _{i+1}(X) - \lambda _{i}(X) | {{\,\textrm{LE}\,}}(X)=\gamma ] \le \psi \,, \end{aligned}$$

for all $$0\le i\le L-1$$.

#### Proof

In the case that *X* is killed upon hitting *W*, see [30, Proof of Claim 3.2].

When *X* is killed at $T_\zeta -1$ where $T_\zeta$ is an independent geometric random variable with mean $\zeta >1$, the proof can be deduced from the previous claim. Indeed, we add a new vertex $\rho$ to *G* and edges $(\rho ,u)$ for every $u\in G$ with weights on them so that the probability to visit $\rho$ from every vertex in a single step is equal to $1/\zeta$ for any $u\in G$. Call the resulting network $G^*$. A random walk on $G^*$ started from $v\in G$ and terminated when hitting $\rho$ has the same distribution as a random walk on *G* with geometric killing time. $\square$

### Mixing times

Recall the definition of the uniform mixing time above Assumption 1.4. It follows that for every $t\ge t_{\textrm{mix}}$ we have that

7 $$\begin{aligned} \frac{\pi (v)}{2} \le {\mathbb {P}}_u(X_t = v) \le 2\pi (v) \,, \end{aligned}$$

where $X_t$ is the random walk and $\pi (v)$ is the stationary measure on *G* satisfying

$$\begin{aligned} \pi (v) = \frac{\deg (v)}{\sum _{u\in G}\deg (u)}. \end{aligned}$$

Even though in this paper we mainly use the uniform mixing time as defined in (2) we also use a more classical version of distance between probability measures on finite sets. Recall that the **total variation distance** between two probability measures on $\mu$ and $\nu$ on a finite set *X* is defined by

$$\begin{aligned} d_{\text {TV}}(\mu ,\nu ) = \max _{A \subset X} |\mu (A)-\nu (A)| \,. \end{aligned}$$

It is a standard fact (see [27, Section 4.5]) that if $t \ge kt_{\textrm{mix}}$, then for any vertex *x*

8 $$\begin{aligned} d_{\text {TV}}(p_t(x,\cdot ),\pi (\cdot )) \le 2^{-k} \,. \end{aligned}$$

### Capacity

The *capacity* of a set of vertices quantifies how difficult it is for a random walk to hit the set. It is a crucial notion when one wishes to analyze the behavior of Wilson’s algorithm. Let $\{Y_i\}_{i\ge 0}$ be a random walk on *G* and for $U \subset V(G)$, let $\tau _U = \inf \{i \ge 0: Y_i \in U\}$. Given $k \ge 0$ we define the ${\textbf{k}}$**-capacity** of *U* by $\textrm{Cap}_k(U) = {\mathbb {P}}_{\pi }\!\left( \tau _U \le k\right)$. If $W \subset V(G)$ is another subset of vertices we define the **relative** ${\textbf{k}}$**-capacity** $\textrm{Cap}_k(W, U) = {\mathbb {P}}_{\pi }\!\left( \tau _W \le k, \tau _W \le \tau _U\right)$. Note that the relative capacity is *not* symmetric in *W*, *U*.

We will see later that the capacities of certain subsets determine the expected volumes of balls in ${{\,\textrm{UST}\,}}(G)$. Here we collect some useful facts about the capacity. By the union bound, when *G* is balanced with parameter *D* we always have the upper bound

9 $$\begin{aligned} \textrm{Cap}_k(V) \le k\pi (V) \le \frac{k D|V|}{n}. \end{aligned}$$

The capacity is defined for the lazy simple random started at stationarity. When *k* is significantly larger than the mixing time, the starting vertex does not make much difference as the following claim shows.

#### Claim 2.3

Let *G* be a connected balanced graph. Let $u\in V$, let $U\subseteq V$ be nonempty, let $r=r(n)\gg \log (n)\cdot t_{\textrm{mix}}(G)$ and assume that $t=t(n)$ is a sequence so that $t(n) \sim r(n)$. Then, for large enough *n*,

$$\begin{aligned} {\mathbb {P}}_u(\tau _U<t) \ge \frac{1}{3}{{\,\textrm{Cap}\,}}_r(U). \end{aligned}$$

#### Proof

See [30, Claim 1.4]. $$\square $$

We will also use the following lemma.

#### Lemma 2.4

Let *G* be a connected regular graph. Let $W\subset U$ be subsets of vertices and $k,s,m\ge 0$. Assume that

$$\begin{aligned} \textrm{Cap}_k(W, U) \ge s \,. \end{aligned}$$

Then we can find at least $L= \lfloor s/(m+kD/n) \rfloor$ disjoint subsets $A_1,\ldots , A_L$ of *W* such that

$$\begin{aligned} m \le \textrm{Cap}_k(A_j, U ) \le m + {kD \over n} \,, \end{aligned}$$

for all $$j=1,\ldots , L$$.

#### Proof

We first observe that if *A* is a subset of *W* and $v \in W {\setminus } A$ then by (9) we have that

$$\begin{aligned} \textrm{Cap}_k( A \cup \{v\}, U ) \le \textrm{Cap}_k(A, U ) + {kD \over n}. \end{aligned}$$

Secondly, we observe that if $A_1,\ldots , A_{L'}$ are disjoint sets so that $\cup _{j=1}^{L'} A_j = W$, then (since $W \subset U$)

$$\begin{aligned} \textrm{Cap}_k(W, U) = \sum _{j=1}^{L'} \textrm{Cap}_k(A_j, U ) \,. \end{aligned}$$

With these two observations in place, we now perform an iterative construction of the subsets. We add vertices from *W* to $A_1$ until the first time that $\textrm{Cap}_k(A_1, U) \ge m$. By the first observation we have that $\textrm{Cap}_k(A_1, U) \le m + kD/n$. Then we add vertices from $W\setminus A_1$ to $A_2$ until the first time that $\textrm{Cap}_k(A_2, U) \ge m$ and so forth. By the second observation we deduce that we can continue this way until at least $L = \left\lfloor \frac{s}{m + \frac{kD}{n}} \right\rfloor$, concluding the proof. $\square$

In order to obtain useful lower bounds on the capacity, we state a well-known relationship between the capacity of a set *A* and the Green kernel summed over *A*. Given a set $A \subset G$ and $k \in {\mathbb {N}}$ we define

10 $$\begin{aligned} M^{(k)}(A) = \sum _{x, y \in A} G^{(k)}(x,y), \end{aligned}$$

where $G^{(k)}(x,y) = {\mathbb {E}}_x \left[ \sum _{i=0}^k \mathbb {1}\{X_i = y\}\right]$. This is useful due to the following relation between this quantity and capacity.

#### Lemma 2.5

Let *G* be a connected balanced graph with parameter *D* on *n* vertices. For all $A \subset G$,

$$\begin{aligned} {{\,\textrm{Cap}\,}}_k (A) \ge \frac{k|A|^2}{2D^3n M^{(k)}(A)}. \end{aligned}$$

#### Proof

The proof is the same as that of [10, Theorem 2.2], but instead considering a stationary starting point distributed according to $\pi$, using the measure $\mu (x) = \frac{ \mathbb {1}\{x \in A\} \pi (x)}{\pi (A)}$, and noting that

1. $$ G^{(k)}(\pi , x) \ge \frac{k}{Dn}$$ for all $$x \in G$$,
2. $$\sum _{x, y \in A} G^{(k)}(x,y) \frac{\pi (x)\pi (y)}{\pi (A)^2} \le \frac{D^2\,M^{(k)}(A)}{|A|^2}$$.

$$\square $$

The following bound on ${\mathbb {E}}\left[ M^{(k)} (P)\right]$ where *P* is a random walk path will be useful.

#### Lemma 2.6

Let *G* be a connected balanced graph with parameter *D* on *n* vertices. Let *m* and *k* be two positive integers and let *P* be a random walk path of length *m* started at $v \in V(G)$. Then

$$\begin{aligned} {\mathbb {E}}\left[ M^{(k)}(P) \right] \le 2mD \sum _{t=0}^{m+k} (t+1)\sup _{u\in V(G)} p_t(u,u) \,. \end{aligned}$$

#### Proof

The proof goes by the same argument as in [19, Lemma 5.6]. $\square$

Furthermore, in order to lower bound the relative capacity, we define the ${\textbf{k}}$**-closeness** of two sets *U* and *W* by

11 $$\begin{aligned} \textrm{Close}_k (U,W) = {\mathbb {P}}_{\pi }\!\left( \tau _{U}< k, \tau _{W} < k\right) \,. \end{aligned}$$

It follows from [34, Lemma 5.2] together with (9) that on any finite connected balanced graph *G*, if $W = X[0,T]$ where *X* is a random walk on *G* started at stationarity, and *T* is a stopping time, then for any set $U \subset G$,

12 $$\begin{aligned} {\mathbb {E}}\left[ \textrm{Close}_k (U, W) \right] \le \frac{4D{\mathbb {E}}[T]k \textrm{Cap}_k(U)}{n} \le \frac{4D^2k^2 |U| {\mathbb {E}}T}{n^2}. \end{aligned}$$

Lastly, recall the two bubble sums defined in Sect. 2.1. One of the uses of the capacity is to bound such bubble sums.

#### Claim 2.7

Let $\{ G_n \}$ be a sequence of graphs satisfying Assumption 1.5 and let $W\subset G_n$ be a set of vertices such that $\textrm{Cap}_{\sqrt{n}}(W) \ge c$, then

$$\begin{aligned} B_{W}(G) \le \theta + \frac{18D}{c^2}. \end{aligned}$$

#### Proof

This follows by exactly the same proof as in [30, Claim 3.14]. $\square$

#### Claim 2.8

Let $\{ G_n \}$ be a sequence of graphs satisfying Assumption 1.5 and let $\zeta > 0$ be given. Then

$$\begin{aligned} {\mathcal {B}}_{\zeta ^{-1} n^{1/2}}(G_n) \le \theta + 2D\zeta ^{-2}. \end{aligned}$$

#### Proof

Take any $v \in G_n$. Then, similarly to [30, Claim 3.14], since $\sum _{t=0}^{\infty }(t+1) \left( 1 - x \right) ^t = x^{-2}$ and using (7),

$$\begin{aligned}&{\mathcal {B}}_{\zeta ^{-1} n^{1/2}}(G_n) = \sum _{t=0}^{\infty }(t+1) \textbf{p}_{}^t(v,v) \left( 1 - \zeta n^{-1/2} \right) ^t \\&\quad \le \theta + \frac{2D}{n}\sum _{t=\sqrt{n}}^{\infty }(t+1) \left( 1 - \zeta n^{-1/2} \right) ^t \le \theta + 2D\zeta ^{-2}. \end{aligned}$$

$$\square $$

### Stochastic domination properties

The ${{\,\textrm{UST}\,}}$ enjoys the negative correlation property, i.e., the probability that an edge *e* is such that $e \in {{\,\textrm{UST}\,}}(G)$ conditioned on $f\in {{\,\textrm{UST}\,}}(G)$ for some other edge *f* is no more than the unconditional probability. Moreover, Feder and Mihail showed that for every increasing event ${\mathcal {A}}$ that ignores *f*, the probability of ${\mathcal {A}}$ given $f\in {{\,\textrm{UST}\,}}(G)$ is no more than the unconditional probability. This led to the following result.

#### Lemma 2.9

[29, Lemma 10.3] Let *G* be a connected subgraph of a finite connected graph *H*. Then, ${{\,\textrm{UST}\,}}(G)$ stochastically dominates ${{\,\textrm{UST}\,}}(H)\cap E(G)$.

The same proof leads to a slightly more generalized version.

#### Lemma 2.10

Let (*G*, *w*) be a weighted network and suppose that $(H,w')$ is a network such that $V(G) \subseteq V(H)$ and that for every edge (*v*, *u*) with $w((v,u)) \ne 0$ we have $w((v,u)) = w'((v,u))$. Then, ${{\,\textrm{UST}\,}}(G)$ stochastically dominates ${{\,\textrm{UST}\,}}(H)\cap E(G)$.

Later in the paper, we will apply Lemma 2.10 in the following context. To study ${{\,\textrm{UST}\,}}(G)$ using Wilson’s algorithm, it will sometimes be convenient to add an extra vertex to *G* called the sun, and for every vertex $v \in G$ add an extra edge from *v* to the sun. We give well-chosen weights to these new edges and call the new graph the **sunny graph**. Lemma 2.10 tells us that the UST of the sunny graph, intersected with *E*(*G*), is stochastically dominated by ${{\,\textrm{UST}\,}}(G)$. This idea was previously used in [40] and [34].

We will also make use of the following well-known lemma. Here *G*/*A* denotes the graph obtained from *G* by identifying all vertices in *A* with a single vertex.

#### Lemma 2.11

[29, Exercise 10.8] Let (*G*, *w*) be a finite network. Let $A\subseteq B$ be two sets of vertices. Then, ${{\,\textrm{UST}\,}}(G/A)$ stochastically dominates ${{\,\textrm{UST}\,}}(G/B)$.

Lastly, let *W* be a set of vertices, and let $A_1$ and $A_2$ be disjoint subsets of *W*. In what follows we consider ${{\,\textrm{UST}\,}}(G/W)$. Given an integer *k* and $j\in \{1,2\}$, let $I_j(k)$ denote the vertices of *G* that are connected to *W* in ${{\,\textrm{UST}\,}}(G/W)$ by a path of length *k* such that the last edge on the path to *W* is an edge that one of its original endpoints belonged to $A_j$ (including $A_j$ itself). Also, let $X_j = X_j(k) = |I_j(k)|$.

#### Claim 2.12

Let *G* be a finite connected graph, take any $k\ge 1$ and let $W, A_1, A_2$ be as above. Then, for ${{\,\textrm{UST}\,}}(G/W)$ and for every $M>0$,

$$\begin{aligned} {\mathbb {E}}\left[ X_2 \big \vert X_1 \le M\right] \ge {\mathbb {E}}\left[ X_2\right] . \end{aligned}$$

#### Proof

We will first show that for every $v\in G$, the events $\{X_1 > M\}$ and $\{v\in I_2\}$ are negatively correlated. Fix some $v\in G$ such that $v\in I_2$ has positive probability. Condition on $v\in I_2$ and on $\gamma _2$, the path from *v* to $A_2$. The ${{\,\textrm{UST}\,}}$ conditioned on *W* and $\gamma _2$ has the distribution of ${{\,\textrm{UST}\,}}(G/(W\cup \gamma _2))$. Hence, by Lemma 2.11 we have that ${{\,\textrm{UST}\,}}(G/(W\cup \gamma _2))$ is dominated by ${{\,\textrm{UST}\,}}(G/W)$. As $\{X_1 > M\}$ is an increasing event that ignores $\gamma _2$ we have that

$$\begin{aligned} {\mathbb {P}}\left( X_1> M \mid v \in I_2(k), \gamma _2 \right) \le {\mathbb {P}}(X_1 > M). \end{aligned}$$

Then by averaging over $\gamma _2$ and taking complements we obtain

$$\begin{aligned} {\mathbb {P}}\left( X_1 \le M \mid v \in I_2(k) \right) \ge {\mathbb {P}}(X_1 \le M). \end{aligned}$$

Therefore, inverting using Bayes’ rule, we have for every *v* with ${\mathbb {P}}(v\in I_2(k)) >0$ that

$$\begin{aligned} {\mathbb {P}}(v\in I_2(k) \mid X_1 \le M) \ge {\mathbb {P}}(v \in I_2(k)). \end{aligned}$$

Summing over *v* yields the result. $\square$

## The Lower Mass Bound

The starting point of the proof of Theorem 1.6 is the work of Peres and Revelle [34].

### Theorem 3.1

[34, Theorem 1.2] Let $\{G_n\}$ be a sequence of graphs satisfying Assumption 1.4 and let ${\mathcal {T}}_n$ be ${{\,\textrm{UST}\,}}(G_n)$. Denote by $d_{{\mathcal {T}}_n}$ the graph distance on ${\mathcal {T}}_n$ and by $({\mathcal {T}},d,\mu )$ the CRT. Then there exists a sequence $\{\beta _n\}$ satisfying $0<\inf _n \beta _n \le \sup _n \beta _n < \infty$ such that the following holds. For fixed $k \ge 1$, if $\{x_1,\ldots , x_k\}$ are uniformly chosen independent vertices of $G_n$, then the distances

$$\begin{aligned} \frac{d_{{\mathcal {T}}_n}(x_i,x_j)}{\beta _n \sqrt{n}} \end{aligned}$$

converge jointly in distribution to the ${k \atopwithdelims ()2}$ distances in ${\mathcal {T}}$ between *k* i.i.d. points drawn according to $\mu$.

For the proof of Theorem 1.6 we take the same sequence $\beta _n$ guaranteed to exist by the theorem above. As we shall see in Sect. 6, the convergence of Theorem 3.1 is equivalent to what is known as Gromov-weak convergence, which does not imply GHP convergence. For example, imagine that we modify ${{\,\textrm{UST}\,}}(K_n)$ by adding a single path of length $\sqrt{n}$ to the vertex labelled 1. Then the result of Theorem 3.1 would still hold, but the sequence no longer converges to the CRT in the GHP topology (it now converges to the CRT attached to a path of length 1 and zero measure).

In order to close this gap in this abstract theory, Athreya, Löhr and Winter [7, Theorem 6.1] introduced the *lower mass bound* condition and proved that this condition together with Gromov-weak convergence is in fact equivalent to GHP convergence; we discuss this further in Sect. 6. The main effort in this paper is proving that the lower mass bound holds under Assumption 1.5 (and hence under Assumption 1.4); this is the content of the following theorem.

### Theorem 3.2

Let $\{ G_n \}$ be a sequence of graphs satisfying Assumption 1.5 and let ${\mathcal {T}}_n$ be ${{\,\textrm{UST}\,}}(G_n)$. For a vertex $v\in {\mathcal {T}}_n$ and some $r\ge 0$ we write $B_{{\mathcal {T}}_n}(v,r) = \{u: d_{{\mathcal {T}}_n}(v,u)\le r\}$ where $d_{{\mathcal {T}}_n}$ is the intrinsic graph distance metric on ${\mathcal {T}}_n$. Then for any $c>0$ and any $\delta >0$ there exists $\varepsilon >0$ such that for all $n\ge 1$,

$$\begin{aligned} {\mathbb {P}}\big ( \exists v \in {\mathcal {T}}_n: |B_{{\mathcal {T}}_n}(v, c\sqrt{n})|\le \varepsilon n \big ) \le \delta . \end{aligned}$$

In other words, the sequence of random variables $\big \{\max _v \{n |B_{{\mathcal {T}}_n}(v, c\sqrt{n})|^{-1}\}\big \}_n$ is tight.

In the rest of this section we prove Theorem 3.2, delegating parts of the proof to Sect. 4 and Sect. 5. For the rest of this section as well as Sect. 4, $\{ G_n \}$ is a sequence of graphs satisfying Assumption 1.5 and ${\mathcal {T}}_n$ is ${{\,\textrm{UST}\,}}(G_n)$.

### Bootstrap argument

The main difficulty in Theorem 3.2 is that it is global; that is, it requires a lower tail bound on the volumes of the balls around *all* vertices simultaneously. As a first attempt, one might hope to obtain a local tail bound for the ball around a single vertex and strengthen this to a global tail bound using a naive union bound; however, since $\frac{1}{n}|B_{{\mathcal {T}}_n}(v, c\sqrt{n})|$ is expected to have a non-trivial scaling limit, the local bound ${\mathbb {P}}\big (|B_{{\mathcal {T}}_n}(v, c\sqrt{n})|\le \varepsilon n \big )$ for fixed $\varepsilon$ and *c* will not decay to zero with *n* and therefore there is no hope to succeed just by taking a union bound over all *n* vertices.

Instead we refine a union bound approach as follows. Our strategy is to bound ${\mathbb {P}}\big (|B_{{\mathcal {T}}_n}(v, c\sqrt{n})|\le \varepsilon n \big )$ as a function of $\varepsilon$, and use a bootstrap argument to obtain a weaker (yet sufficient) global bound. The idea is to use the observation that if there is one vertex $x \in {\mathcal {T}}_n$ such that |*B*(*x*, *r*)| is small, then either $|B(x, \frac{r}{2})|$ is also small, or otherwise there are many vertices $v \in B(x, \frac{r}{2})$ such that $|B(v, \frac{r}{2})|$ is small. Provided that we have sufficiently stronger tail bounds for the latter events compared to the former, this allows us to define a sequence of events of the form

13 $$\begin{aligned} \left\{ \left| B\left( x, \frac{r}{2^\ell }\right) \right| \le {\varepsilon \over 4^\ell } \left( \frac{r}{2^\ell }\right) ^2 \right\} , \end{aligned}$$

for which we obtain stronger tail bounds as $\ell$ increases. Therefore we can iterate this until the probability is upper bounded by $o\left( \frac{1}{n}\right)$, at which point we will apply the union bound and conclude the proof.

Thus, our goal will be to iteratively improve the tail bounds on (13), where *x* is a fixed vertex (our graphs are transitive so the choice of *x* does not matter) and $\ell = 0,\ldots , N_n$ where $N_n$, the number of iterations, will be chosen suitably as we now explain.

Since we will use Wilson’s algorithm to sample branches in ${{\,\textrm{UST}\,}}(G_n)$, it will be important in our arguments in Sect. 4 that the radius $\frac{c \sqrt{n}}{2^l}$ we consider at each step is significantly longer than the mixing time of a random walk on $G_n$. Therefore, we require that $\frac{c \sqrt{n}}{2^{N_n}} \gg n^{\frac{1}{2} - \alpha }$ (recall the constant $\alpha$ from Assumption 1.5), so the number of iterations $N_n$ can be at most of order $\log n$. We will see in the proof of Theorem 3.2 that for this bootstrap argument to work with only $\log n$ steps, it will be convenient to obtain tail bounds for (13) that are sub-polynomial in $\varepsilon$.

A natural strategy to bound the probability of the event in (13) is to first sample a single branch joining *x* to a pre-defined root of ${{\,\textrm{UST}\,}}(G_n)$, consider the volumes of balls in subtrees attached to this branch close to *x*, and show that the sum of these volumes is very unlikely to be too small. This strategy almost gives sufficiently strong tail decay, but there is one step at which the tail decay is not sub-polynomial. This problem arises in the first step since there is a probability of order $\varepsilon$ that the path joining *x* to a root vertex is of length less than $\sqrt{\varepsilon n}$.

This is not a fundamental problem since if this path is short, then it means we just picked a short branch when longer branches to different roots were available. However, it is not convenient to condition on picking a long branch to a well-chosen root since this conditioning reveals too much information about ${{\,\textrm{UST}\,}}(G_n)$, which makes it difficult to control other properties of the branch, primarily its capacity and the capacity of its subsets. It is also inconvenient (though probably possible) to continue choosing a few more branches until we reach a certain length.

The simplest way we found to circumvent this issue is to first sample a branch $\Gamma _n$ between two uniformly chosen vertices of $G_n$ and perform the bootstrap argument discussed above conditioned on $\Gamma _n$ and the event that it is a “nice” path, a property we will define later that will include, amongst others, the requriement that $\Gamma _n$ is not too short. Then, using Wilson’s algorithm we may sample other branches of ${{\,\textrm{UST}\,}}(G_n)$ by considering loop-erased random walks terminated at $\Gamma _n$; thus $\Gamma _n$ can be thought of as the backbone of ${{\,\textrm{UST}\,}}(G_n)$, and provided $\Gamma _n$ is sufficiently long we can sample the branch from *x* to $\Gamma _n$ and consider its extension into $\Gamma _n$ to make it longer if necessary. With this modified definition of a branch, it is then possible to prove a conditional sub-polynomial tail bound in $\varepsilon$ for (13), and then to prove Theorem 3.2 by decomposing according to whether $\Gamma _n$ is “nice” or not.

Throughout the rest of this paper, and in accordance with Theorem 3.2, we fix $c>0$ to be a small enough parameter and $\varepsilon >0$ which can also be chosen to be small enough depending on *c* and set

14 $$\begin{aligned} N_n = \frac{\alpha }{10} \log _2 n, \qquad r = c\sqrt{n}, \end{aligned}$$

and for any scale $\ell \in \{0,\ldots , N_n\}$

15 $$\begin{aligned} r_\ell = \frac{r}{2^\ell }, \qquad \varepsilon _\ell = \frac{\varepsilon }{4^\ell }, \qquad k_\ell = \varepsilon _\ell ^{1/2} r_\ell . \end{aligned}$$

#### Theorem 3.3

Let $\{ G_n \}$ be a sequence of graphs satisfying Assumption 1.5, let ${\mathcal {T}}_n$ be ${{\,\textrm{UST}\,}}(G_n)$ and denote by $\Gamma _n$ the unique path between two independent uniformly chosen vertices. Then for any $\delta >0$ there exist $c', \varepsilon '>0$ such that for all $c\in (0,c')$ and all $\varepsilon \in (0,\varepsilon ')$ there exists $N = N(\delta , c, \varepsilon )$ such that for any $n\ge N$ we have that, with probability at least $1-\delta$,

(I) $$\textrm{Cap}_{\sqrt{n}} (\Gamma _n) \ge 2Dc$$,
(II) For any scale $\ell \in \{0,\ldots , N_n\}$ and subsegment $I\subseteq \Gamma _n$ with $|I| = r_\ell /3$ we have that
  $$\begin{aligned} \textrm{Cap}_{k_\ell }(I, \Gamma _n) \ge \frac{\varepsilon _{\ell }^{1/6} k_\ell r_\ell }{n} \,, \end{aligned}$$
(III) $$|\Gamma _n| \le \varepsilon ^{-\frac{1}{10}}\sqrt{n}$$.

#### Definition 3.4

For the rest of this paper, given *c* and $\varepsilon$ as above we denote by ${\mathcal {E}}_{n,c,\varepsilon }$ the intersection of the events in (I), (II), (III) of the above theorem.

#### Remark 3.5

The reader may notice that although *c* is fixed in Theorem 3.2 and (14), it is now treated as a variable parameter in Theorem 3.3. This is intentional since we need $|\Gamma _n| \ge c\sqrt{n}$ to overcome the problem of branch length mentioned above, and we cannot ensure this with high probability when *c* is fixed; only when it is small. To prove Theorem 3.2, we start with a fixed *c*, but our first step is to reduce it if necessary so that the statement of Theorem 3.3 holds as well. We will then prove the theorem with this smaller value of *c*. This poses no problem since the assertion of Theorem 3.2 with smaller *c* is stronger; this is also discussed in the proof of Theorem 3.2.

Next we assume that ${\mathcal {E}}_{n,c,\varepsilon }$ holds for some positive *c* and $\varepsilon$, and let *x* be a vertex of $G_n$. Let $\Gamma _x$ denote the loop-erasure of the random walk path starting at *x* and stopped when it hits $\Gamma _n$ (if $x\in \Gamma _n$ then $\Gamma _x$ is empty). For an integer $s\in (0,c\sqrt{n})$ we denote by $\Gamma _x^s$ the prefix of $\Gamma _x$ of length *s* as long as $|\Gamma _x|\ge s$; otherwise, i.e. if $|\Gamma _x|<s$, we denote by $\Gamma _x^s$ the prefix of the path in ${{\,\textrm{UST}\,}}(G_n)$ from *x* to one of the two endpoints of $\Gamma _n$ such that this path has length at least *s*. This is possible since by part (I) of Theorem 3.3 and (9), if ${\mathcal {E}}_{n,c,\varepsilon }$ holds, then $|\Gamma _n| \ge 2c\sqrt{n}$. If the two endpoints of $\Gamma _n$ can be used, we choose one in some arbitrary predefined manner. In Sect. 4.3 we will prove the following.

#### Theorem 3.6

Let $\{ G_n \}$ be a sequence of graphs satisfying Assumption 1.5 and let ${\mathcal {T}}_n$ be ${{\,\textrm{UST}\,}}(G_n)$. Denote by $\Gamma _n$ the unique path between two independent uniformly chosen vertices and for a vertex $x \in G_n$ and $s>0$ let $\Gamma ^s_x$ be as described above. Then for any $c >0$ there exist $\varepsilon '>0$ and a constant $a > 0$ such that for every $\varepsilon \in (0,\varepsilon ')$ there exists $N=N(c,\varepsilon )$ such that for any $n\ge N$ and any $\ell \in \{0,\ldots , N_n\}$ we have for every $x\in G_n$

$$\begin{aligned} {\mathbb {P}}\left( \textrm{Cap}_{k_\ell }\left( \Gamma _x^{5r_\ell /6}, \Gamma _n \cup \Gamma _x\right) \le \frac{\varepsilon _\ell ^{\frac{1}{6}} k_\ell r_\ell }{n} \ \textrm{and} \ {\mathcal {E}}_{n,c,\varepsilon }\right) \le e^{-a (\log \varepsilon _\ell ^{-1})^2}. \end{aligned}$$

Given these two estimates we are now ready to proceed with the proof of Theorem 3.2. Our strategy is as follows. On the event

16 $$\begin{aligned} \left\{ \textrm{Cap}_{k_\ell }\left( \Gamma _x^{5r_\ell /6}, \Gamma _n \cup \Gamma _x \right) \ge \frac{\varepsilon _\ell ^{\frac{1}{6}} k_\ell r_\ell }{n}\right\} , \end{aligned}$$

we can condition on $\Gamma _n$ and $\Gamma _x$ and then apply Lemma 2.4 with $m = \frac{2^{11}D^{3}er_{\ell }k_{\ell }\varepsilon _{\ell }^{\frac{1}{2}}}{n}$ and $s=\frac{r_{\ell }k_{\ell }\varepsilon _{\ell }^{\frac{1}{6}}}{n}$ to obtain $L = \frac{\varepsilon _{\ell }^{-1/3}}{2^{12}D^{3}e}$ disjoint subintervals $A_1, \ldots , A_L$ of $\Gamma _x^{5r_\ell /6}$ such that

$$\begin{aligned} \textrm{Cap}_{k_\ell }(A_j, \Gamma _n \cup \Gamma _x) \ge \frac{2^{11}D^{3}e\varepsilon _{\ell }r_{\ell }^2}{n} \end{aligned}$$

for all $j=1,\ldots , L$. Moreover, since the cardinality of $\cup _{j=1}^L A_j$ is at most $\frac{5r_{\ell }}{6}$, the number of *j*’s such that $|A_j| \ge 2^{13}D^{3}e\varepsilon _{\ell }^{1 \over 3} r_{\ell }$ is at most $\frac{5}{6\cdot D^{3} 2^{13}e} \varepsilon _{\ell }^{-\frac{1}{3}}$. Hence the number of *j*’s for which $|A_j| \le (2^{13}D^{3}e)\varepsilon _{\ell }^{1 \over 3} r_{\ell }$ is at least $(2^{13}D^{3}e)^{-1}\varepsilon _{\ell }^{-\frac{1}{3}}$; we relabel the sets so that $A_i$ for $i=1,\ldots , (2^{13}D^{3}e)^{-1}\varepsilon _{\ell }^{-\frac{1}{3}}$ have this upper bound on their size and forget about the other sets.

Our aim will be to test each of the intervals $\{A_i\}$ in turn to see if the trees hanging on $A_i$ contribute at least $r_{\ell }^2 \varepsilon _{\ell }$ to $B(x, r_{\ell })$. We will test these intervals *conditionally* on $\Gamma _x \cup \Gamma _n$ and on the outcome of the previous tests. Here we encounter a significant difficulty since the failure of some past tests introduces a complicated conditioning which we cannot access directly by contracting some edges.

To overcome this difficulty we proceed as follows. Conditioned on $\Gamma _n \cup \Gamma _x \subset {{\,\textrm{UST}\,}}(G_n)$, we contract $\Gamma _n \cup \Gamma _x$ to a single vertex (still remembering the original edge-set) to form the graph $G_n / (\Gamma _n \cup \Gamma _x)$. By the UST spatial Markov property [9, Proposition 4.2], we have that ${{\,\textrm{UST}\,}}(G_n)$ is distributed as the union of $\Gamma _n \cup \Gamma _x$ and the ${{\,\textrm{UST}\,}}$ of this new graph. Before proceeding, we then add a new vertex called **the sun**, denoted by $\odot$, to the graph $G_n / (\Gamma _n \cup \Gamma _x)$, and add an edge from every vertex to the sun with weight chosen so that a lazy random walk on $G_n \cup \{\odot \} / (\Gamma _n \cup \Gamma _x)$ will always jump to the sun at the next step with probability $\frac{1}{k_{\ell }}$. Then, we identify the sun with $\Gamma _n \cup \Gamma _x$, remembering the edges emanating from the sun. This ensures that when we run Wilson’s algorithm on the remaining graph, rooted at the contracted vertex, random walks will always be killed when they hit the sun, so typically they only run for time of order $k_{\ell }$.

On the graph $G_n/(\{\odot \} \cup \Gamma _n \cup \Gamma _x)$ we will often say “**hit** ${\textbf{A}}$” when *A* is a subset of $\{\odot \} \cup \Gamma _n \cup \Gamma _x$. The meaning of hitting *A* in the graph $G_n/(\{\odot \} \cup \Gamma _n \cup \Gamma _x)$ is to hit $\{\odot \} \cup \Gamma _n \cup \Gamma _x$ by traversing an edge whose original endpoint belonged in *A*. In some cases it will be convenient to start a random walk at a uniform vertex *U* in the original graph $G_n$, and project the start point onto $G_n/(\{\odot \} \cup \Gamma _n \cup \Gamma _x)$; in this case “hit *A*" also includes the event $U \in A$.

By Lemma 2.10, conditionally on $\Gamma _n \cup \Gamma _x$, we have that ${{\,\textrm{UST}\,}}(G_n / (\Gamma _n \cup \Gamma _x))$ stochastically dominates ${{\,\textrm{UST}\,}}(G_n / (\{\odot \} \cup (\Gamma _n \cup \Gamma _x)) \cap E(G_n / (\Gamma _n \cup \Gamma _x))$. Therefore, we can couple the two ${{\,\textrm{UST}\,}}$s together such that every edge *e* not adjacent to the sun in ${{\,\textrm{UST}\,}}(G_n / (\{\odot \} \cup (\Gamma _n \cup \Gamma _x))$ also appears in ${{\,\textrm{UST}\,}}(G_n / (\Gamma _n \cup \Gamma _x))$. When we expand $\{\odot \} \cup \Gamma _n \cup \Gamma _x$ in ${{\,\textrm{UST}\,}}(G_n / (\{\odot \} \cup (\Gamma _n \cup \Gamma _x))$ and then remove $\odot$ and its incident edges, we obtain several connected components, one of which contains *x*. By stochastic domination, the component containing *x* is a subset of ${{\,\textrm{UST}\,}}(G_n)$. Therefore, let $B^{\odot }(x, r_{\ell })$ denote the set of vertices connected to *x* by a path of length at most $r_{\ell }$ that does not intersect the sun after expanding $\{\odot \} \cup \Gamma _n \cup \Gamma _x$ in the sunny graph. By stochastic domination, if we can prove a lower tail bound for $B^{\odot }(x, r_{\ell })$ on the sunny graph, it automatically transfers to a lower tail bound for $B_{{\mathcal {T}}_n}(x, r_{\ell })$ on the original graph.

Recall that, given $\Gamma _n \cup \Gamma _x$, each of the $A_i$’s defined above is a subset of $\Gamma _n \cup \Gamma _x$. When working on the graph $G_n/(\{\odot \} \cup \Gamma _n \cup \Gamma _x)$, we let $I_i(k_{\ell })$ be the set of vertices connected to the contracted vertex in ${{\,\textrm{UST}\,}}(G_n/(\{\odot \} \cup \Gamma _n \cup \Gamma _x)$ by a path of length at most $k_{\ell }$, such that the last edge on this path has an endpoint in $A_i$. Note that this is equivalent to being connected to $A_i$ by a path of length at most $k_{\ell }$ not touching $\Gamma _n\cup \Gamma _x$ after expanding the path and separating $\odot$ to obtain a subset of ${{\,\textrm{UST}\,}}(G_n)$. We also include $A_i$ in $I_i(k_{\ell })$ and set $X_i = X_i(k_{\ell }) = |I_i(k_{\ell })|$.

Let $B^{\odot }_j= \{ \sum _{i=1}^j X_i \le 16\varepsilon _{\ell }r_{\ell }^2\}$ and (for notational convenience) interpret $B^{\odot }_0$ as an almost sure event. In Sect. 5 we will prove the following lemma.

#### Lemma 3.7

Conditionally on $\Gamma _x \cup \Gamma _n$, let $B^{\odot }_j$ be as defined above on the graph $G_n/(\{\odot \} \cup \Gamma _n \cup \Gamma _x)$. Then for each $j\le (2^{13}D^{3}e)^{-1}\varepsilon _{\ell }^{-\frac{1}{3}}$,

$$\begin{aligned} {\mathbb {P}}\left( B^{\odot }_j\big \vert B^{\odot }_{j-1}, \Gamma _n \cup \Gamma _x, \textrm{Cap}_{k_\ell }(\Gamma _x^{5r_{\ell }/6}, \Gamma _n \cup \Gamma _x ) \ge \frac{ r_{\ell }k_{\ell }\varepsilon _{\ell }^{\frac{1}{6}}}{n} \right) \le 1 - \frac{1}{160D^{3}e}\varepsilon _{\ell }^{1/6}. \end{aligned}$$

This has the following immediate corollary.

#### Corollary 3.8

Let $\{ G_n \}$ be a sequence of graphs satisfying Assumption 1.4 and let ${\mathcal {T}}_n, \Gamma _n$ and $\Gamma _x$ be as in the previous theorem. Then for any $c>0$, any $\varepsilon > 0$, all *n* large enough and any $\ell \in \{0,\ldots , N_n\}$, we have

$$\begin{aligned} {\mathbb {P}}\left( |B_{{\mathcal {T}}_n}(x,r_\ell )| \le 16 r_\ell ^2 \varepsilon _\ell \,\,, \,\, \textrm{Cap}_{k_\ell }(\Gamma _x^{5r_\ell /6}, \Gamma _n \cup \Gamma _x) \ge \frac{\varepsilon _\ell ^{\frac{1}{6}} k_\ell r_\ell }{n}\right) \le \exp \left\{ -b {\varepsilon _{\ell }^{-1/6}} \right\} \,, \end{aligned}$$

where $$b=(5e^2D^{6}2^{18})^{-1}$$.

#### Proof

Given that $\textrm{Cap}_{k_\ell }(\Gamma _x^{5r_\ell /6}, (\Gamma _n \cup \Gamma _x) {\setminus } \Gamma _x^{5r_\ell /6}) \ge \frac{\varepsilon _\ell ^{\frac{1}{6}} k_\ell r_\ell }{n}$, we can condition on $\Gamma _n \cup \Gamma _x$ and obtain intervals $(A_j)_{j=1}^{(2^{13}D^{3}e)^{-1}\varepsilon _{\ell }^{-1/3}}$ on the graph $G_n/(\{\odot \} \cup \Gamma _n \cup \Gamma _x)$ as described above. Applying Lemma 3.7, we then deduce that

$$\begin{aligned}&{\mathbb {P}}\left( |B^{\odot }(x,r_\ell )| \le 16 r_\ell ^2 \varepsilon _\ell \big \vert \textrm{Cap}_{k_\ell }(\Gamma _x^{5r_\ell /6} , \Gamma _n \cup \Gamma _x ) \ge \frac{\varepsilon _\ell ^{\frac{1}{6}} k_\ell r_\ell }{n}, \,\, \Gamma _n \cup \Gamma _x \right) \\&\quad \le \prod _{j=1}^{(2^{13}e)^{-1}\varepsilon _{\ell }^{-1/3}} {\mathbb {P}}\left( B^{\odot }_j\big \vert B^{\odot }_{j-1}, (\Gamma _n \cup \Gamma _x), \textrm{Cap}_{k_\ell }(\Gamma _x^{5r_{\ell }/6} , \Gamma _n \cup \Gamma _x ) \ge \frac{ r_{\ell }k_{\ell }\varepsilon _{\ell }^{\frac{1}{6}}}{n} \right) \\&\quad \le \left( 1-\frac{1}{160D^{3}e}\varepsilon _{\ell }^{1/6}\right) ^{(2^{13}D^{3}e)^{-1}\varepsilon _{\ell }^{-1/3}} \le \exp \left\{ -b {\varepsilon _{\ell }^{-1/6}} \right\} . \end{aligned}$$

To conclude, we average over $\Gamma _n \cup \Gamma _x$, then transfer this result from $B^{\odot }(x,r_\ell )$ in ${{\,\textrm{UST}\,}}(G_n/(\{\odot \} \cup \Gamma _n \cup \Gamma _x)$ to $B_{{\mathcal {T}}_n}(x,r_\ell )$ in ${{\,\textrm{UST}\,}}(G_n)$ using the stochastic domination result of Lemma 2.10, as explained above. $\square$

We now have all the tools to prove Theorem 3.2.

#### Proof of Theorem 3.2

Let $\delta >0$. We define the events

$$\begin{aligned} A_{\ell }&= \left\{ \exists x \in {\mathcal {T}}_n: \left| B\left( x, r_\ell \right) \right| \le {\varepsilon _\ell r_\ell ^2} \text { and } \left| B\left( x, r_{\ell +1}\right) \right| \ge \varepsilon _{\ell +1} r_{\ell +1}^2 \right\} , \\ B_{\ell }&= \left\{ \exists x \in {\mathcal {T}}_n: \left| B\left( x, r_\ell \right) \right| \le \varepsilon _\ell r_\ell ^2 \right\} \, , \end{aligned}$$

for $\ell \in \{0,\ldots , N_n\}$. We decompose by writing

17 $$\begin{aligned} {\mathbb {P}}\left( \exists x \in {\mathcal {T}}_n: |B(x, c \sqrt{n})| \le \varepsilon c^2 n\right)&\le {\mathbb {P}}\left( \lnot {\mathcal {E}}_{n,c,\varepsilon }\right) + \left( \sum _{\ell =0}^{N_n-1} {\mathbb {P}}\left( {\mathcal {E}}_{n,c,\varepsilon }\cap A_{\ell } \right) \right) \nonumber \\&\quad + {\mathbb {P}}\left( {\mathcal {E}}_{n,c,\varepsilon }\cap B_{N_n} \right) . \end{aligned}$$

In what follows we will show that given $\delta >0$ we can find $\varepsilon$ and *c* small enough and *N* large enough so that the sum above is at most $3\delta$. This yields the required assertion of the theorem since the quantity ${\mathbb {P}}(\exists x \in {\mathcal {T}}_n: |B(x, c \sqrt{n})| \le \varepsilon n)$ is non-decreasing as *c* decreases.

We first apply Theorem 3.3 and find $\varepsilon$ and *c* small enough and *N* large enough (depending on $\delta$) that the first term is at most $\delta$ for all $n \ge N$. To control the second term in (17) we note that if $A_{\ell }$ occurs, then $\left| B\left( v, r_{\ell +1}\right) \right| \le \varepsilon _{\ell } r_{\ell }^2=16\varepsilon _{\ell +1} r_{\ell +1}^2$ for all $v \in B\left( x, r_{\ell +1}\right)$, and the number of such *v* is at least $\varepsilon _{\ell +1}r_{\ell +1}^2$. Therefore using Theorem 3.6, Corollary 3.8 and Markov’s inequality, we have for all *n* large enough that

18 $$\begin{aligned} \begin{aligned}&\sum _{l=0}^{N_n-1} {\mathbb {P}}\left( {\mathcal {E}}_{n,c,\varepsilon }\cap A_{\ell } \right) \\&\le \sum _{l=0}^{N_n-1} {\mathbb {P}}\left( {\mathcal {E}}_{n,c,\varepsilon }\ \textrm{and} \ \big | \left\{ v \in {\mathcal {T}}_n: \left| B\left( v, r_{\ell +1}\right) \right| \le 16\varepsilon _{\ell +1} r_{\ell +1}^2 \right\} \big | \ge \varepsilon _{\ell +1}r_{\ell +1}^2 \right) \\&\le n \sum _{l=0}^{N_n-1} \varepsilon _{\ell +1}^{-1}r_{\ell +1}^{-2} \big ( e^{-a (\log \varepsilon _\ell ^{-1})^2} + e^{-b\varepsilon _\ell ^{-\frac{1}{6}}} \big ) \, . \end{aligned} \end{aligned}$$

By making $\varepsilon$ smaller and *N* larger if necessary we can guarantee that the term in the parenthesis on the right hand side is at most $\varepsilon _\ell ^{10}$ for all $n \ge N$, and hence that

$$\begin{aligned} n \sum _{l=0}^{N_n-1} \varepsilon _{\ell +1}^{-1}r_{\ell +1}^{-2} \big ( e^{-a (\log \varepsilon _\ell ^{-1})^2} + e^{-b\varepsilon _\ell ^{-\frac{1}{6}}} \big ) \le c^{-2} \varepsilon ^9 \sum _{l=0}^{\infty }2^{-16\ell }. \end{aligned}$$

This shows that the sum can be smaller than $\delta$ as long as $\varepsilon$ is small enough and *N* is large enough.

Finally, for the third term we recall that $r_{N_n} = c n^{\frac{1}{2} - \frac{\alpha }{10}}$ and $\varepsilon _{N_n} = \varepsilon n^{-\frac{\alpha }{5}}$, and use Theorem 3.6, Corollary 3.8 and the union bound to bound

$$\begin{aligned} {\mathbb {P}}\left( {\mathcal {E}}_{n,c,\varepsilon }\cap B_{N_n} \right) \le n\left( e^{-a \log ^2 (\varepsilon ^{-1}n^{\alpha /5})} + e^{-b\varepsilon ^{-\frac{1}{6}}n^{\alpha /30} } \right) \,, \end{aligned}$$

which tends to 0 as $n\rightarrow \infty$, so it is smaller than $\delta$ as long as *n* is large enough. Provided *n* is sufficiently large, we have therefore bounded (17) by $3\delta$, concluding the proof. (We can then reduce $\varepsilon$ if necessary so that the bound holds for all $n\ge 1$.) $\square$

## Proofs of Theorems 3.3 and 3.6

In this section we prove Theorem 3.3 and Theorem 3.6. Due to the results of [30], this essentially boils down to proving only capacity estimates. In both cases, we will bound capacity using Lemma 2.5. In Sect. 4.1 we prove two claims that we later use in the proofs of Theorem 3.3 and Theorem 3.6 in Sect. 4.2 and Sect. 4.3 respectively.

### Two claims

In what follows we take $z = 1/20$ (there is in fact some flexibility in the choice of *z*, but this value is compatible with our choices elsewhere), we fix some $\psi >0$ (this will be a bound for the bubble sum), and for $\ell \ge 0$ we define two parameters $K_{\ell }$ and $L_{\ell }$ by

19 $$\begin{aligned} K_{\ell }= \frac{z}{3\psi } \varepsilon _{\ell }^{-\frac{2z}{3}}\log \varepsilon _{\ell }^{-1}, \quad L_{\ell }:= \frac{1}{2} K_{\ell }\varepsilon _{\ell }^{5z/3} r_{\ell }= \frac{z}{6\psi } \varepsilon _{\ell }^{z}(\log \varepsilon _{\ell }^{-1}) r_{\ell }. \end{aligned}$$

The notation $K_{\ell }$ will be mainly used in Claim 4.1 to show that there are at least $K_{\ell }$ consecutive LERW intervals, each of length $\varepsilon _{\ell }^{\frac{5z}{3}} r_{\ell }$ and of total length larger than $L_{\ell }$, in the $r_{\ell }$-prefix deriving from “fairly short" intervals of the original random walk. In Claim 4.2 we will consider random walk intervals of length $L_{\ell }$, and show that they contain “good" subintervals, meaning that these subintervals have good capacity and closeness properties. The lengths of these subintervals will be chosen so that eventually we can tie everything up by showing that the random walk segment corresponding to the $K_{\ell }$ consecutive LERW intervals must contain a good random walk subinterval, which in turn must contain one of the $K_{\ell }$ consecutive LERW intervals that we started with, and we will deduce that this final LERW interval has good capacity.

We also assume that $\{G_n\}$ is a sequence of graphs satisfying Assumption 1.5. As mentioned above, our first claim shows that with very high probability any loop-erased trajectory (that has bounded bubble-sum) has a rather long subinterval which is derived from a (relatively) short segment of a random walk trajectory (which in turn will have a long subinterval with good $M^{(k)}$ and closeness values by the subsequent claim).

#### Claim 4.1

Fix $\psi >0$ and $c>0$. There exists $\varepsilon ' >0$ such that for every $\varepsilon \in (0,\varepsilon ')$ there exists *N* such that for all $n\ge N$ and for all scales $\ell \in \{0,\ldots ,N_n\}$ the following holds. Let *X* be a random walk on $G_n$ which is bubble-terminated (see Definition 2.1) with bubble-sum bounded by $\psi$ and let $\Gamma$ be its loop erasure. Also fix $j\in {\mathbb {N}}$.

Then with probability at least $1-\exp \left( -\frac{\varepsilon _{\ell }^{-\frac{z}{3}}}{\log (1/\varepsilon _{\ell })}\right)$ either

$$\begin{aligned} |\Gamma | < \frac{jr_{\ell }}{24} \,, \end{aligned}$$

or there exists $t \in [(j-1)r_{\ell }/24,jr_{\ell }/24]$ such that for all integers $1 \le m \le K_{\ell }$,

$$\begin{aligned} \lambda _{t+m \varepsilon _{\ell }^{\frac{5z}{3}} r_{\ell }}(X) - \lambda _{t+(m - 1) \varepsilon _{\ell }^{\frac{5z}{3}} r_{\ell }}(X) \le \varepsilon _{\ell }^{z}r_{\ell }\,. \end{aligned}$$

#### Proof

We set $Q_\ell =\varepsilon _{\ell }^{-5z/3}$. On the event that $|\Gamma | \ge jr_{\ell }/24$, we divide $\Gamma [(j-1)r_{\ell }/24,jr_{\ell }/24]$ into $Q_\ell /24$ consecutive disjoint subintervals of length $r_{\ell }/Q_\ell$. For $m \in \{1,\ldots , Q_\ell /24\}$ we say that the *m*-th interval is good if

$$\begin{aligned} \lambda _{\frac{(m+1)r_{\ell }}{Q_\ell }(X)} - \lambda _{\frac{mr_{\ell }}{Q_\ell }}(X) \le \frac{r_{\ell }}{Q_\ell ^{3/5}} = \varepsilon _{\ell }^{z}r_{\ell }\,. \end{aligned}$$

As we assumed that the bubble sum is bounded by $\psi$, it follows from Claim 2.2 that conditioned on $\Gamma$ and the event $\{|\Gamma | \ge jr_{\ell }/24\}$, the collection of events that the *m*-th interval is good are independent. Furthermore, by Claim 2.2 and Markov’s inequality the probability of each such event is at least

$$\begin{aligned} 1-\frac{\psi }{Q_\ell ^{2/5}}. \end{aligned}$$

Hence, the probability that a sequence of $K_{\ell }$ disjoint consecutive intervals are all good is at least

$$\begin{aligned} (1- \psi Q_\ell ^{-2/5})^{K_{\ell }} \ge \varepsilon _{\ell }^{2z/3} \,, \end{aligned}$$

where we used the inequality $1-x \ge e^{-2x}$ valid for $x>0$ small enough. Since there are $\frac{Q_\ell }{24}$ intervals in total, we can form $\frac{Q_\ell }{24K_{\ell }}$ disjoint runs of $K_{\ell }$ consecutive intervals. Since the events are independent conditionally on $\Gamma$, we deduce that the probability that none of these runs contain only good intervals is at most

$$\begin{aligned} \left( 1-\varepsilon _{\ell }^{2z/3}\right) ^{\frac{Q_\ell }{24K_{\ell }}} \le \exp \left( -\frac{\varepsilon _{\ell }^{2z/3 -5z/3}}{24K_{\ell }}\right) \le \exp \left( -\varepsilon _{\ell }^{-{z \over 3}} \over \log \varepsilon _{\ell }^{-1}\right) , \end{aligned}$$

where in the last inequality we used the fact that $\frac{z}{3 \psi } \le \frac{1}{24}$. $\square$

For the next claim recall the definitions of $M^{(k)}$ in (10) and of $\textrm{Close}_k(U,V)$ in (11). We show that with very high probability, any random walk interval of length of order $L_{\ell }$ has a slightly shorter subinterval of length of order $\varepsilon _{\ell }^z r_{\ell }$, such that its value of $M^{(k)}$ and its closeness to the rest of the path are very close to their expected values given by Lemma 2.6 and (12). This is done by finding many well separated intervals and employing the fast mixing of the graph to obtain independence.

#### Claim 4.2

Fix $\psi >0$ and $c>0$. There exists $\varepsilon ' >0$ such that for every $\varepsilon \in (0,\varepsilon ')$ there exists *N* such that for all $n\ge N$ and for all scales $\ell \in \{0,\ldots ,N_n\}$ the following holds. Let *X* be a random walk on $G_n$, started from stationarity. Let $M>0$ and fix some interval $I \subset [0,M\sqrt{n}]$ with $|I|= L_{\ell }$ (note that |*I*| depends on $\psi$). Also let $W \subset G_n$ be fixed. Then with probability at least $1-2e^{-\frac{z^2}{16\psi }(\log \varepsilon _{\ell }^{-1})^{2}}$ there exists a subinterval $J = [t_J^-, t_J^+] \subset I$ such that

1. $$|J| = 2 \varepsilon _{\ell }^{z}r_{\ell }$$,
2. $$M^{(k_{\ell })}(X[J]) \le \frac{r_{\ell }}{4D^3}$$
3. $$\textrm{Close}_{k_{\ell }} \left( X[J], X\left[ t_J^+ + \frac{r_{\ell }}{24}, M\sqrt{n}\right] \cup X[0, t_J^- - \frac{r_{\ell }}{24}]\right) \le \frac{ r_{\ell }k_{\ell }^2\,M}{n^{1.5}}$$.
4. $$\textrm{Close}_{k_{\ell }} (X[J], W) \le \frac{ r_{\ell }k_{\ell }^2 |W|}{n^{2}}$$.

#### Proof

We write $I=[t_I^-,t_I^+]$ and then further subdivide *I* into $\frac{z}{15\psi }\log \varepsilon _{\ell }^{-1}$ segments of length $2\varepsilon _{\ell }^{z}r_{\ell }$ separated by buffers of length $\frac{1}{2}\varepsilon _{\ell }^{z}r_{\ell }$, that is, we set

20 $$\begin{aligned} I_j = [t_j^-, t_j^+):= {\left[ t_I^- + \frac{5}{2}j\varepsilon _{\ell }^{z}r_{\ell }+ \frac{1}{4}\varepsilon _{\ell }^{z}r_{\ell },t_I^- + \frac{5}{2}(j+1)\varepsilon _{\ell }^{z}r_{\ell }- \frac{1}{4}\varepsilon _{\ell }^{z}r_{\ell }\right) } \end{aligned}$$

for each non-negative integer $j \le \frac{z}{15\psi }\log \varepsilon _{\ell }^{-1}$. It will be important soon that the length of the buffers satisfy $\frac{1}{4}\varepsilon _{\ell }^{z}r_{\ell }\ge \frac{1}{4}\varepsilon _{N_n}^{z}r_{N_n} \gg n^{\frac{2\alpha }{3}} t_{\textrm{mix}}$ for all *n* large enough by Assumption 1.5, (14) and (15). We also set

$$\begin{aligned} X^{\textbf{avoid}}_{I}= X\left[ 0, t_I^- - \frac{r_{\ell }}{36}\right) \cup X{\left[ t_I^+ + \frac{r_{\ell }}{36}, M\sqrt{n}\right) }. \end{aligned}$$

We condition on $X^{\textbf{avoid}}_{I}$ and define for each *j* the event

$$\begin{aligned} {\mathcal {E}}_{j}= & {} \left\{ M^{(k_{\ell })}(X[I_j]) \le r_{\ell }/4D^3 \ \text {and} \ \textrm{Close}_{k_{\ell }}\left( X[I_j], X^{\textbf{avoid}}_{I}\right) \right. \\{} & {} \quad \left. \le \frac{r_{\ell }k_{\ell }^2 M}{n^{3/2}} \ \text {and} \ \textrm{Close}_{k_{\ell }}\left( X[I_j], W \right) \le \frac{r_{\ell }k_{\ell }^2|W|}{n^2}\right\} . \end{aligned}$$

Note that by definition of *I*, we have that $t_j^- - t_I^- \le \frac{r_{\ell }}{72}$ and $t_I^+ - t_j^+ \le \frac{r_{\ell }}{72}$ for all *j* and all $\ell$ provided $\varepsilon$ is small enough. Thus,

$$\begin{aligned} \textrm{Close}_{k_{\ell }}(X[I_j],X^{\textbf{avoid}}_{I}) \ge \textrm{Close}_{k_{\ell }}\left( X[I_j], X\left[ 0, t_j^- - \frac{r_{\ell }}{24}\right] \cup X\left[ t_j^+ + \frac{r_{\ell }}{24}, M\sqrt{n}\right] \right) . \end{aligned}$$

Hence ${\mathcal {E}}_{j}$ implies that the interval $I_j$ satisfies the conditions $(1)-(4)$. Note that the events $\{{\mathcal {E}}_{j}\}_j$ are not independent, but that was why we introduced the buffers. Let $\{Y_j\}_{j \le \frac{z}{15\psi }\log \varepsilon _{\ell }^{-1}}$ be independent random walks started from stationarity and run for time $2\varepsilon _{\ell }^{z}r_{\ell }$, set

$$\begin{aligned} {\mathcal {E}}^{\textrm{ind}}_{j}= & {} \left\{ M^{(k_{\ell })}(Y_j) \le r_{\ell }/4D^3 \ \text {and} \ \textrm{Close}_{k_{\ell }}\left( Y_j, X^{\textbf{avoid}}_{I}\right) \right. \\{} & {} \quad \left. \le \frac{r_{\ell }k_{\ell }^2 M}{n^{3/2}} \ \text {and} \ \textrm{Close}_{k_{\ell }}\left( Y_j, W \right) \le \frac{r_{\ell }k_{\ell }^2|W|}{n^2}\right\} \,, \end{aligned}$$

and note that conditioned on $X^{\textbf{avoid}}_{I}$ the events $\{{\mathcal {E}}^{\textrm{ind}}_{j}\}_j$ are independent. Now by Lemma 2.6 and Assumption 1.5 we have (provided $\varepsilon <1$ and $c< 1/6$, for example) that

$$\begin{aligned} {\mathbb {E}}\left[ M^{(k_{\ell })}(Y_j)\right] \le 4D\theta \varepsilon _{\ell }^{z}r_{\ell }\,. \end{aligned}$$

Since $|X^{\textbf{avoid}}_{I}| \le M\sqrt{n}$, it also follows from (12) that

$$\begin{aligned} {\mathbb {E}}\left[ \textrm{Close}_{k_{\ell }} \left( Y_j, X^{\textbf{avoid}}_{I}\right) \big |X^{\textbf{avoid}}_{I}\right] \le \frac{8\varepsilon _{\ell }^{z}r_{\ell }k_{\ell }^2 MD^2}{n^{3/2}} \,, \end{aligned}$$

and that

$$\begin{aligned} {\mathbb {E}}\left[ \textrm{Close}_{k_{\ell }} \left( Y_j, W\right) \right] \le \frac{8\varepsilon _{\ell }^{z}r_{\ell }k_{\ell }^2|W|D^2}{n^{2}} \,. \end{aligned}$$

Consequently, by Markov’s inequality and independence we get that

21 $$\begin{aligned} {\mathbb {P}}\left( \text {none of } \{{\mathcal {E}}^{\textrm{ind}}_{j}\} \text { occur} \big | X^{\textbf{avoid}}_{I}\right) \le \left( (16+16\theta )D^{4}\varepsilon _{\ell }^{{z}}\right) ^{\frac{z}{15\psi }(\log \varepsilon _{\ell }^{-1})} \le e^{-\frac{ z^2}{16\psi }(\log \varepsilon _{\ell }^{-1})^{2}}\, , \end{aligned}$$

as long as $\varepsilon$ is small enough depending on $\theta$ and *D*. To conclude, note that as long as *n* is large enough, we can couple the independent walks $\{Y_j\}$ and $\{X[I_j]\}$ so that

22 $$\begin{aligned} {\mathbb {P}}\left( \exists j: X[I_j] \ne Y_j\right) \le \frac{z \log (1/\varepsilon _{\ell })}{15\psi }2^{-n^{\frac{2\alpha }{3}}} \le e^{-\frac{z^2}{16\psi }(\log \varepsilon _{\ell }^{-1})^{2}} \, . \end{aligned}$$

Indeed, assume we coupled the first $j-1$ pairs and condition on all these pairs. By the Markov property and since the buffers between distinct $I_j$’s are longer than $n^{\frac{2\alpha }{3}}t_{\textrm{mix}}$, the starting point of $X[I_j]$ is $2^{-n^{\frac{2\alpha }{3}}}$ close in total variation distance to the stationary distribution by (8). Therefore, we may couple it to the first vertex of $Y_j$ so that they are equal with probability at least $1-2^{-n^{\frac{2\alpha }{3}}}$ (see for instance [27, Proposition 4.7]). Moreover, once their starting points are coupled, we can run the walks together so that they remain coupled for the remaining $2\varepsilon _{\ell }^{z}r_{\ell }$ steps. Hence (22) holds for large enough *n* and we combine with (21) in a union bound to conclude that

$$\begin{aligned} {\mathbb {P}}\left( \text {none of } \{{\mathcal {E}}_{j}\} \text { occur} \big | X^{\textbf{avoid}}_{I}\right) \le 2 e^{-\frac{z^2}{16\psi }(\log \varepsilon _{\ell }^{-1})^{2}} \,. \end{aligned}$$

$$\square $$

### Proof of Theorem 3.3

As mentioned in Sect. 2.4, to sample $\Gamma _n$ we will use a coupling with the sunny graph $G_n^* = G_n^*(\zeta )$ introduced in [34], obtained from $G_n$ by adding an extra vertex $\rho _n$ known as the sun, and connecting it to every vertex in $v \in G_n$ with an edge of weight $\frac{ (\deg v) \zeta }{\sqrt{n}-\zeta }$ (so that the probability of jumping to $\rho _n$ at any step is $\zeta n^{-1/2}$). It follows from Lemma 2.10 that the graph ${{\,\textrm{UST}\,}}(G_n^*){\setminus }\{\rho _n\}$ obtained from the UST of $G_n^*$ by removing $\rho _n$ and its incident edges is stochastically dominated by the UST of $G_n$. Therefore, there is a coupling between ${{\,\textrm{UST}\,}}(G_n)$ and ${{\,\textrm{UST}\,}}(G_n^*)$ such that ${{\,\textrm{UST}\,}}(G_n^*){\setminus }\{\rho _n\} \subset {{\,\textrm{UST}\,}}(G_n)$; moreover if ${\widetilde{\Gamma }}_n^*$ denotes the path between $u$ and $v$ in ${{\,\textrm{UST}\,}}(G_n^*)$, then $\Gamma _n = {\widetilde{\Gamma }}_n^*$ in this coupling provided that $\rho _n \notin {\widetilde{\Gamma }}_n^*$.

Note that this sunny graph is *different* to the sunny graph used in the statements of Lemma 3.7 and Corollary 3.8. As outlined in Sect. 3.1, Lemma 3.7 and Corollary 3.8 refer to later stages of the overall proof strategy.

Consequently, it will be convenient to work with a path $\Gamma _n^* = \Gamma _n^*(\zeta )$ sampled as follows. Let *T* and $T'$ be two independent geometric random variables with mean $\zeta ^{-1} n^{1/2}$. Given *T*, let *X* be a random walk run for $T-1$ steps started from $u \in G_n$ and let ${{\,\textrm{LE}\,}}(X)$ be its loop erasure. Given $T'$ and *X*, run $X'$, a random walk started from $v \in G_n$ and terminate $X'$ after $T'$ steps. Write $T_X$ for the minimum between $T'$ and the first hitting time of *X*. Let $\Gamma _n^*$ be the path between (*u*, *v*) in ${{\,\textrm{LE}\,}}(X)\cup {{\,\textrm{LE}\,}}(X'[0,T_X])$, if such a path exists (we will see below that this happens with high probability). Otherwise, let $\Gamma _n^* = \emptyset$.

#### Lemma 4.3

For every $\delta >0$ there exists $\zeta >0$ such that for all large enough *n* there exists a coupling of $\Gamma _n$ and $\Gamma _n^*(\zeta )$ such that $\Gamma _n = \Gamma _n^*(\zeta )$ and is non-empty with probability at least $1-\delta$.

#### Proof

For every path $\Gamma$ from *u* to *v* on $G_n^*(\Gamma )$ let $H(\Gamma ) = \Gamma$ if $\rho _n\notin \Gamma$ and $H(\Gamma ) = \emptyset$ if $\rho _n\in \Gamma$. Run Wilson’s algorithm on the graph $G_n^*(\zeta )$ initiated at the points $\rho _n, u$ and then *v*, and note that $\tau _{\rho _n}$ (the hitting time of $\rho _n$) is a geometric random variable, and moreover that given $\tau _{\rho _n}$, the walk until time $\tau _{\rho _n}$ is distributed as a random walk on $G_n$. Consequently, $H({\widetilde{\Gamma }}_n^*)$ has the distribution of $\Gamma _n^*$.

By the discussion above, we can find a coupling of $(\Gamma _n, {\widetilde{\Gamma }}_n^*)$ where these paths are equal whenever $\rho _n \notin {\widetilde{\Gamma }}_n^*$. Under this coupling, we have that $(\Gamma _n, {\widetilde{\Gamma }}_n^*, H({\widetilde{\Gamma }}_n^*))$ are all equal with probability ${\mathbb {P}}(\rho _n \notin {\widetilde{\Gamma }}_n^*)$. As $H({\widetilde{\Gamma }}_n^*)$ has the law of $\Gamma _n^*$, this is in fact a coupling of $\Gamma _n$ and $\Gamma _n^*$ where the paths are equal with probability ${\mathbb {P}}(\rho _n \notin {\widetilde{\Gamma }}_n^*)$. By [30, Claim 2.9], this probability tends to 1 as $\zeta \rightarrow 0$. For the final part of the claim, note that $\Gamma _n^*$ is clearly non-empty on this good event. $\square$

Note that using the sun to sample $\Gamma _n^*(\zeta )$ in this way allows us to sample the path between *u* and *v* using roughly $\sqrt{n}$ steps of a simple random walk, so that Claim 4.2 can be applied (with high probability). On the other hand, if we were to naively sample this path by erasing loops of a simple random walk from *u* to *v*, it would require approximately *n* steps. In addition it allows us to control the bubble sum of such a random walk, using Claim 2.8, so that Claim 4.1 can be applied. We can therefore use this construction to prove Theorem 3.3 as follows.

#### Proof of Theorem 3.3

Our main effort is to show that part (II) holds with high probability. Indeed, that part (I) and (III) occur with probability at least $1-\delta$ as long as $\varepsilon >0$ and $c>0$ are small enough is a consequence of [30, Theorem 1.1 and Theorem 2.1].

Let $\delta >0$. We appeal to Proposition 4.3 and obtain $\zeta >0$ so that ${\mathbb {P}}\left( \Gamma _n \ne \Gamma _n^*(\zeta )\right) \le \delta /4$. Denote by ${\mathcal {B}}$ the event of part (II) of Theorem 3.3. For the rest of the proof, we think of $\delta$ and $\zeta$ as fixed, we set $\psi = \theta + 2D\zeta ^{-2}$ and decrease both *c* and $\varepsilon$ as follows until we eventually obtain that ${\mathbb {P}}({\mathcal {B}}^c)\le \delta$.

Recall that $\Gamma _n^*(\zeta )$ is generated using two independent random walks with geometric killing time which we denote *X* and $X'$. Setting $M = \frac{8}{\zeta \delta }$, we can thus write

$$\begin{aligned} {\mathbb {P}}({\mathcal {B}}^c) \le {\mathbb {P}}\left( \Gamma _n \ne \Gamma _n^*(\zeta )\right) + {\mathbb {P}}\left( |X|+|X'| \ge M\sqrt{n}\right) + {\mathbb {P}}\left( |X|+|X'| \le M\sqrt{n} \ \text {and} \ {\mathcal {B}}^c\right) . \end{aligned}$$

The first event has probability at most $\frac{\delta }{4}$ by the above. Since $M = \frac{8}{\zeta \delta }$, the probability of the second event is also bounded by $\delta /4$ by Markov’s inequality. For the third term, first decrease *c* if necessary so it is less than $\frac{1}{2M}$ (this will be useful at the end of the proof), then let $\ell \le N_n$ be a fixed scale and let $I\subset \Gamma _n^*(\zeta )$ be some segment with $|I| = r_{\ell }/3$. It therefore has at least $r_{\ell }/ 6$ vertices either on ${{\,\textrm{LE}\,}}(X)$ or on ${{\,\textrm{LE}\,}}(X')$ and hence contains at least one interval of the form ${{\,\textrm{LE}\,}}(X)[(j-2)r_{\ell }/24, (j+1)r_{\ell }/24]$ or ${{\,\textrm{LE}\,}}(X')[(j-2)r_{\ell }/24, (j+1)r_{\ell }/24]$ (that is, an interval of the form $[(j-1)r_{\ell }/24, jr_{\ell }/24]$ plus two buffers of length $\frac{r_{\ell }}{24}$ both before and after the interval) for some $j \le {24\,M \sqrt{n} \over r_{\ell }}$.

For the rest of the proof, recall that $z=1/20$ and the definitions of $K_{\ell }$ and $L_{\ell }$ from (19). Since Assumption 1.5 holds, we deduce from Claim 2.8 that *X* and $X'$ are bubble-terminated random walks with bubble sum bounded by $\psi$. Hence we may apply Claim 4.1 and the union bound to learn that the probability that there exists a scale $\ell$ and *j* as above such that the event of Claim 4.1 does not hold for *X* or $X'$ is at most

$$\begin{aligned} \sum _{\ell =0}^{\infty }\frac{M\sqrt{n}}{r_{\ell }/24}\exp \left( -\frac{\varepsilon _{\ell }^{-\frac{z}{3}}}{\log (1/\varepsilon _{\ell })}\right)= & {} \frac{24M}{c} \sum _{\ell =0}^{\infty } 2^\ell \cdot \exp \left( -\frac{\varepsilon _{\ell }^{-\frac{z}{3}}}{\log (1/\varepsilon _{\ell })}\right) \\= & {} \frac{24M}{c}\sum _{\ell =0}^{\infty } 2^\ell \cdot \exp \left( -\frac{\varepsilon ^{-\frac{z}{3}} 4^{\frac{\ell z}{3}}}{\log (4^\ell \varepsilon ^{-1})}\right) \,, \end{aligned}$$

which can be made to be smaller than $\delta /4$ by decreasing $\varepsilon$ appropriately. Thus we may assume without loss of generality that *I* contains an interval of the form ${{\,\textrm{LE}\,}}(X)[(j-2)r_{\ell }/24, (j+1)r_{\ell }/24]$ for some *j* that we fix henceforth, and that there exists a time $t\in [(j-1)r_{\ell }/24, jr_{\ell }/24]$ such that for all integers *m* satisfying $1 \le m \le K_{\ell }$ we have

23 $$\begin{aligned} \lambda _{t+m \varepsilon _{\ell }^{\frac{5z}{3}} r_{\ell }}(X) - \lambda _{t+(m - 1) \varepsilon _{\ell }^{\frac{5z}{3}} r_{\ell }}(X) \le \varepsilon _{\ell }^{z}r_{\ell }. \end{aligned}$$

We write $$X[t_1,t_2)$$ for the corresponding part of *X*, that is, we set $$t_1 = \lambda _{t}(X)$$ and $$t_2 = \lambda _{t+2L_{\ell }}(X)$$. It holds by construction that

24 $$\begin{aligned} t_2 - t_1 \ge 2L_{\ell }\quad \text {and} \quad t_2 \le M\sqrt{n} \,. \end{aligned}$$

We now apply the union bound using Claim 4.2 with $W=X'[0, M\sqrt{n}]$ to get that the probability that there exists a scale $\ell$ and $i\le \frac{M\sqrt{n}}{L_{\ell }}$ such that $(1)-(4)$ of Claim 4.2 do *not* hold for the interval $\left[ \left( i-1\right) L_{\ell }, i L_{\ell }\right]$ is at most

$$\begin{aligned}&\sum _{\ell =0}^{\infty } \frac{M\sqrt{n}}{L_{\ell }} \cdot \exp \left( - \frac{z^2}{16\psi } \left( \log \varepsilon _{\ell }^{-1}\right) ^{2}\right) \\&\quad = \sum _{\ell =0}^{\infty } \frac{6\psi M\sqrt{n}}{z \varepsilon _{\ell }^{z}(\log \varepsilon _{\ell }^{-1}) r_{\ell }} \cdot \exp \left( - \frac{z^2}{16\psi } \left( \log \varepsilon _{\ell }^{-1}\right) ^{2}\right) \\&\quad \le \frac{2M\psi }{3cz} \sum _{\ell =0}^{\infty } \frac{\left( 2\cdot 4^z\right) ^{\ell }}{\varepsilon ^z\log \varepsilon ^{-1}} \left( \frac{\varepsilon }{4^\ell }\right) ^{\log \left( \frac{4^\ell }{\varepsilon }\right) \frac{z^2}{16\psi }}, \end{aligned}$$

which can be made smaller than $\delta /4$ by decreasing $\varepsilon$ appropriately. Therefore we henceforth assume that all such intervals contain a good subinterval satisfying $(1)-(4)$ of Claim 4.2.

Since $[t_1, t_2]$ must contain an interval of the form $\left[ \left( i-1\right) L_{\ell }, i L_{\ell }\right]$ by (24), it now follows that $[t_1,t_2]$ contains a subinterval $J=[t_J^-,t_J^+]$ satisfying conditions $(1)-(4)$ of Claim 4.2 with $W=X'[0, M\sqrt{n}]$. Since

$$\begin{aligned} |J| = 2\varepsilon _{\ell }^{z}r_{\ell }\ge \lambda _{t+m \varepsilon _{\ell }^{\frac{5z}{3}} r_{\ell }}(X) - \lambda _{t+(m - 2) \varepsilon _{\ell }^{\frac{5z}{3}} r_{\ell }}(X) \end{aligned}$$

for each $2 \le m\le K_{\ell }$ by (23), there must exist some $m^* \le K_{\ell }$ such that

$$\begin{aligned} t_J^- \le \lambda _{t+(m^* - 1) \varepsilon _{\ell }^{\frac{5z}{3}} r_{\ell }}(X) < \lambda _{t+m^* \varepsilon _{\ell }^{\frac{5z}{3}} r_{\ell }}(X) \le t_J^+. \end{aligned}$$

We set $A=({{\,\textrm{LE}\,}}(X))_{[t+(m^* - 1) \varepsilon _{\ell }^{\frac{5z}{3}} r_{\ell }, t+m^* \varepsilon _{\ell }^{\frac{5z}{3}} r_{\ell })}$, so that $A \subset I \subset \Gamma _n$, so that $|A|=\varepsilon _{\ell }^{\frac{5z}{3}} r_{\ell }$ and so that $A \subset X[t_J^-, t_J^+]$. Since $M^{(k_{\ell })}(A)$ defined in (10) is monotone in *A* we have that $M^{(k_{\ell })}(A) \le M^{(k_{\ell })}(X[t_J^-, t_J^+]) \le r_{\ell }/4D^3$ by (2) of Claim 4.2. Hence by Lemma 2.5,

$$\begin{aligned} \textrm{Cap}_{k_\ell }(A) \ge \frac{k_{\ell }|A|^2}{2nD^3M^{(k_{\ell })}(A)} \ge \frac{2k_{\ell }r_{\ell }\varepsilon _{\ell }^{\frac{10z}{3 }}}{n} \,. \end{aligned}$$

Due to the buffers of length $r_{\ell }/24$ present in the beginning and ending of *I* it follows that $\left( \Gamma _n^* {\setminus } I\right) \subseteq X[0, t_J^- - r_{\ell }/24] \cup X\left[ t_J^+ + r_{\ell }/24, M\sqrt{n}\right] \cup W$. Hence by $(3)-(4)$ of Claim 4.2 we get

$$\begin{aligned}&\textrm{Close}_{k_{\ell }} \left( A, \Gamma _n^* \setminus I \right) \\&\quad \le \textrm{Close}_{k_{\ell }} \left( X[t_J^-, t_J^+], X[0, t_J^- - r_{\ell }/24] \cup X\left[ t_J^+ + r_{\ell }/24, M\sqrt{n}\right] \cup X' \right) \\&\quad \le \frac{2r_{\ell }k_{\ell }^2M}{ n^{3/2}} = \frac{2\varepsilon _{\ell }^{\frac{10z}{3}}r_{\ell }k_{\ell }}{n} \cdot \frac{M\varepsilon _{\ell }^{\frac{1}{2}-\frac{10z}{3}}r_{\ell }}{\sqrt{n}} \, , \end{aligned}$$

where we used $k_{\ell }= \varepsilon _{\ell }^{1/2}r_{\ell }$ and $|X| + |X'| \le M\sqrt{n}$. Consequently, since we chose $c<\frac{1}{2\,M}$ and $z=1/20$, we can reduce $\varepsilon$ if necessary so that

$$\begin{aligned}&\textrm{Cap}_{k_\ell }\left( I, \Gamma _n^* \right) \\&\quad \ge \textrm{Cap}_{k_\ell }(A) - \textrm{Close}_{k_{\ell }} (A, \Gamma _n^* \setminus I) \ge \frac{2k_{\ell }r_{\ell }\varepsilon _{\ell }^{\frac{10z}{3}}}{n} \left( 1 -\frac{M\varepsilon _{\ell }^{\frac{1}{2}-\frac{10z}{3}}r_{\ell }}{\sqrt{n}} \right) \ge \frac{k_{\ell }r_{\ell }\varepsilon _{\ell }^{\frac{10z}{3}}}{n}, \end{aligned}$$

as required. Finally, to cover the case where *I* is primarily contained in ${{\,\textrm{LE}\,}}(X')$ rather than ${{\,\textrm{LE}\,}}(X)$, note that we can reverse the roles of *X* and $X'$ above to obtain a fourth contribution to the probability of $\frac{\delta }{4}$. This concludes the proof. $\square$

### Proof of Theorem 3.6

We assume that ${\mathcal {E}}_{n,c,\varepsilon }$ holds and let $\ell \le N_n$ be a fixed scale throughout the proof. The proof will involve applications of Claim 4.1 and Claim 4.2; for these we will take $\psi = \theta + \frac{9}{2Dc^2}$, take $M_\ell = \varepsilon _{\ell }^{-1/10}$ and take $W = \Gamma _n$. These three variables will assume these values throughout the proof.

#### Proof

Let *x* be some vertex of $G_n$ and let *X* be a random walk started from *x* and let $\tau _{\Gamma _n}$ denote the time at which *X* hits $\Gamma _n$, so that $\Gamma _x = {{\,\textrm{LE}\,}}(X[0, \tau _{\Gamma _n}])$. We start by upper bounding the time until *X* hits $\Gamma _n$. On the event ${\mathcal {E}}_{n,c,\varepsilon }$ we have from Theorem 3.3 (I) that $\textrm{Cap}_{\sqrt{n}} (\Gamma _n) \ge 2Dc$. It therefore follows from Claim 2.3 that for each $i \ge 1$,

$$\begin{aligned} {\mathbb {P}}\left( X[(i-1) \sqrt{n}, i\sqrt{n}) \cap \Gamma _n \ne \emptyset \big | X[0, (i-1) \sqrt{n}) \cap \Gamma _n = \emptyset \right) \ge \frac{2Dc}{3}. \end{aligned}$$

Consequently, taking a product over $i \le M_\ell$ it follows that

25 $$\begin{aligned} {\mathbb {P}}\left( \tau _{\Gamma _n} > M_\ell \sqrt{n} \right) \le \left( 1 - \frac{2Dc}{3} \right) ^{M_\ell } \le e^{-2DcM_\ell /3}. \end{aligned}$$

Provided that $\varepsilon$ is small enough (depending on *c* and *D* and $\theta$), this is much smaller than the required bound on the probability of Theorem 3.6; hence we work on the event $\{\tau _{\Gamma _n} \le M_\ell \sqrt{n}\}$ for the rest of the proof. Furthermore, if $\{|\Gamma _x| \le \frac{r_{\ell }}{3}\}$, then $\Gamma _x^{5r_{\ell }/6}$ contains a segment $I \subset \Gamma _n$ with $|I| = \frac{r_{\ell }}{3}$ (see the definitions above Theorem 3.6). Hence on the event ${\mathcal {E}}_{n,c,\varepsilon }$ it follows from Theorem 3.3 (II) (which we just proved in the previous subsection) that

$$\begin{aligned} \textrm{Cap}_{k_{\ell }}\left( \Gamma _x^{5r_{\ell }/6}, (\Gamma _n \cup \Gamma _x) \right) \ge \textrm{Cap}_{k_{\ell }} (I, \Gamma _n) \ge \frac{\varepsilon _{\ell }^{1/6} k_{\ell }r_{\ell }}{n}, \end{aligned}$$

so that the tail bound of Theorem 3.6 holds. We therefore also assume that $\{|\Gamma _x| > \frac{r_{\ell }}{3}\}$.

Under ${\mathcal {E}}_{n,c,\varepsilon }$ we have that $\textrm{Cap}_{\sqrt{n}}(\Gamma _n) \ge 2Dc$ and hence by Claim 2.7 we have that $B_{\Gamma _n}(G_n) \le \theta + \frac{9}{2Dc^2}=\psi$, so *X* is bubble-terminated random walk with bubble sum bounded by $\psi$. Now divide $X([0, M_\ell \sqrt{n}])$ into $M_\ell \sqrt{n}( L_{\ell })^{-1}$ disjoint consecutive intervals of length $L_{\ell }$. Also note that $M_\ell \sqrt{n} \le L_{\ell }e^{\frac{z^2}{32 \psi }(\log \varepsilon _{\ell }^{-1})^{2}}$ for all $\ell \le N_n$ provided that $\varepsilon$ is small enough as a function of $\psi$ and *c* (i.e., depending on $\theta$, *D* and *c*). By the union bound and Claim 4.2, provided *n* exceeds some $N(c, \varepsilon )$ the probability that all of these consecutive intervals contain a subinterval satisfying points $(1)-(4)$ of Claim 4.2 is therefore at least

$$\begin{aligned}{} & {} 1 - 2M_\ell \sqrt{n}\left( L_{\ell }\right) ^{-1}e^{-\frac{z^2}{16\psi }(\log \varepsilon _{\ell }^{-1})^{2}} \\{} & {} \quad \ge 1 - 2e^{\frac{z^2}{32\psi }(\log \varepsilon _{\ell }^{-1})^{2}} e^{-\frac{z^2}{16\psi }(\log \varepsilon _{\ell }^{-1})^{2}} = 1 - 2e^{-\frac{z^2}{32\psi }(\log \varepsilon _{\ell }^{-1})^{2}}. \end{aligned}$$

In particular, since *any* interval $I \subset [0, M_\ell \sqrt{n}]$ of length $2L_{\ell }$ must contain an entire consecutive interval of the form above, we deduce that, provided $n \ge N(c, \varepsilon )$,

26 $$\begin{aligned}{} & {} {\mathbb {P}}\left( \forall I \subset [0, M_\ell \sqrt{n}], |I| = 2L_{\ell }: \exists J \subset I \ \textrm{ satisfying } \ (1)-(4) \ \mathrm { of \ Claim~4.2} \right) \nonumber \\{} & {} \quad \ge 1 - 2e^{-\frac{z^2}{32\psi }(\log \varepsilon _{\ell }^{-1})^{2}}. \end{aligned}$$

We next apply Claim 4.1 (recall that $z=1/20$) with $j=1$ to obtain that, provided $n \ge N(c, \varepsilon )$, with probability at least

27 $$\begin{aligned} 1- \exp \left( -\frac{\varepsilon _{\ell }^{-\frac{z}{3}}}{\log (1/\varepsilon _{\ell })}\right) \end{aligned}$$

there exists $t \le \frac{r_{\ell }}{3}$ such that for all $1 \le m \le K_{\ell }$,

28 $$\begin{aligned} \lambda _{t+m \varepsilon _{\ell }^{\frac{5z}{3}} r_{\ell }}(X) - \lambda _{t+(m - 1) \varepsilon _{\ell }^{\frac{5z}{3}} r_{\ell }}(X) \le \varepsilon _{\ell }^{z}r_{\ell }\,. \end{aligned}$$

We write $$X[t_1,t_2)$$ for the corresponding part of *X*, so that $$t_1 = \lambda _{t}(X)$$ and $$t_2 = \lambda _{t+2L_{\ell }}(X)$$. It holds by construction that

29 $$\begin{aligned} 2L_{\ell }\le t_2 - t_1 \,, \end{aligned}$$

and moreover since we assumed that $\{|\Gamma _x| > \frac{r_{\ell }}{3}\}$ and $\{\tau _{\Gamma _n} \le M_\ell \sqrt{n}\}$, we clearly have that $t_2 \le M_\ell \sqrt{n}$. On the event ${\mathcal {E}}_{n,c,\varepsilon }$, it therefore follows from (26) and (29) that the probability that $[t_1,t_2]$ does not contain a subinterval $J=[t_J^-,t_J^+]$ satisfying conditions $(1)-(4)$ of Claim 4.2 is bounded by $2e^{-\frac{z^2 }{32\psi } (\log \varepsilon _{\ell }^{-1})^2}$.

For the rest of the proof we assume that such a *J* exists and that ${\mathcal {E}}_{n,c,\varepsilon }$ holds. By part (1) of Claim 4.2 and (28),

$$\begin{aligned} |J| = 2\varepsilon _{\ell }^{z}r_{\ell }\ge \lambda _{t+m \varepsilon _{\ell }^{\frac{5z}{3}} r_{\ell }}(X) - \lambda _{t+(m - 2) \varepsilon _{\ell }^{\frac{5z}{3}} r_{\ell }}(X) \end{aligned}$$

for each $2 \le m\le K_{\ell }$. Therefore there must exist some $m^* \le K_{\ell }$ such that

$$\begin{aligned} t_J^- \le \lambda _{t+(m^* - 1) \varepsilon _{\ell }^{\frac{5z}{3}} r_{\ell }}(X) < \lambda _{t+m^* \varepsilon _{\ell }^{\frac{5z}{3}} r_{\ell }}(X) \le t_J^+. \end{aligned}$$

Now set $$A=(\Gamma _x)_{[t+(m^* - 1) \varepsilon _{\ell }^{\frac{5z}{3}} r_{\ell }, t+m^* \varepsilon _{\ell }^{\frac{5z}{3}} r_{\ell })}.$$

Note that, by construction, it holds that $A \subset \Gamma _x^{\frac{r_{\ell }}{3}}$, that $|A|=\varepsilon _{\ell }^{\frac{5z}{3}} r_{\ell }$ and that $A \subset X[t_J^-, t_J^+]$. Since $M^{(k_{\ell })}(A)$ as defined in (10) is monotone with respect to *A* we have that $M^{(k_{\ell })}(A) \le M^{(k_{\ell })}(X[t_J^-, t_J^+]) \le r_{\ell }/4D^3$ by (2) of Claim 4.2. Hence by Lemma 2.5,

$$\begin{aligned} \textrm{Cap}_{k_\ell }(A) \ge \frac{k_{\ell }|A|^2}{2nD^3M^{(k_{\ell })}(A)} \ge \frac{2k_{\ell }r_{\ell }\varepsilon _{\ell }^{\frac{10z}{3}}}{n} \,. \end{aligned}$$

Since $t_J^+ \le \lambda _{r_{\ell }/3}(X)$ by construction, and $W=\Gamma _n$, it also follows that

$$\begin{aligned} (\Gamma _n \cup \Gamma _x) \setminus \Gamma _x^{5r_\ell /6} \subset W \cup X[t_J^+ + r_{\ell }/24, M_\ell \sqrt{n}]. \end{aligned}$$

Therefore, since $\textrm{Close}_{k_{\ell }}(\cdot , \cdot )$ is monotone and subadditive in each argument (by definition and the union bound), applying $(3)-(4)$ of Claim 4.2 we deduce that

$$\begin{aligned} \textrm{Close}_{k_{\ell }} \left( A, (\Gamma _n \cup \Gamma _x) \setminus \Gamma _x^{5r_\ell /6} \right)&\le \textrm{Close}_{k_{\ell }} \left( X[t_J^-, t_J^+], X\left[ t_J^+ + r_{\ell }/24, M_\ell \sqrt{n}\right] \right) \\&\quad + \textrm{Close}_{k_{\ell }} \left( X[t_J^-, t_J^+], W \right) \\&\le \frac{r_{\ell }k_{\ell }^2 (M_\ell \sqrt{n}+ |W|)}{ n^{2}} \le \frac{2\varepsilon _{\ell }^{\frac{10z}{3}}r_{\ell }k_{\ell }}{n} \cdot \frac{M_\ell \varepsilon _{\ell }^{\frac{1}{2} - \frac{10z}{3}}r_{\ell }}{\sqrt{n}} \, , \end{aligned}$$

(where we used $k_{\ell }= \varepsilon _{\ell }^{1/2}r_{\ell }$ and $|W| \le \varepsilon _{\ell }^{-1/10} \sqrt{n}= M_{\ell }\sqrt{n}$ on the event ${\mathcal {E}}_{n,c,\varepsilon }$). Consequently, since $z=1/20$, recalling that $M_\ell =\varepsilon _{\ell }^{-1/10}$ and assuming without loss of generality that $c, \varepsilon < 1/2$, we obtain that

$$\begin{aligned} \textrm{Cap}_{k_\ell }\left( \Gamma _x^{r_{\ell }/3}, (\Gamma _n \cup \Gamma _x) \setminus \Gamma _x^{5r_\ell /6} \right)&\ge \textrm{Cap}_{k_\ell }\left( A, (\Gamma _n \cup \Gamma _x) \setminus \Gamma _x^{5r_\ell /6} \right) \\&\ge \textrm{Cap}_{k_\ell }(A) - \textrm{Close}_{k_{\ell }} \left( A, (\Gamma _n \cup \Gamma _x) \setminus \Gamma _x^{5r_\ell /6} \right) \\&\ge \frac{2k_{\ell }r_{\ell }\varepsilon _{\ell }^{\frac{10z}{3}}}{n} \left( 1 -\frac{\varepsilon _{\ell }^{\frac{7}{30}}r_{\ell }}{\sqrt{n}} \right) \ge \frac{k_{\ell }r_{\ell }\varepsilon _{\ell }^{\frac{1}{6}}}{n}. \end{aligned}$$

To summarize, we showed that $\textrm{Cap}_{k_\ell }\left( \Gamma _x^{r_{\ell }/3}, (\Gamma _n \cup \Gamma _x) {\setminus } \Gamma _x^{5r_\ell /6} \right)$ is large enough on the event ${\mathcal {E}}_{n,c,\varepsilon }$ whenever $\{\tau _{\Gamma _n} \le M_\ell \sqrt{n}\}$ and the relevant events in Claim 4.1 and Claim 4.2 occur so that we can find *A* as above. Theorem 3.6 therefore follows on taking a union bound over (25), (26) and (27), choosing $\varepsilon '$ small enough as a function of *c*, *D* and $\theta$ and requiring that *n* is large enough as a function of $\varepsilon ,c,D$ and $\theta$ (since $\psi$ was a function of *c*, *D* and $\theta$). $\square$

## Proof of Lemma 3.7

In this section we prove Lemma 3.7. Throughout we assume that the index *n*, the scale $\ell$ and the paths $\Gamma _n \cup \Gamma _x$ are fixed. We also take the setup of Sect. 3.1, as outlined above Lemma 3.7. This means that we condition on $\Gamma _n \cup \Gamma _x$ and add a sun $\odot$ to the graph $G_n/(\Gamma _n \cup \Gamma _x)$ with weights chosen so that a lazy random walk will jump to the sun at the next step with probability $\frac{1}{k_{\ell }}$. We also assume that the intervals $A_j \subset \Gamma _x$ for $j=1,\ldots , (2^{13}D^{3}e)^{-1}\varepsilon _{\ell }^{-\frac{1}{3}}$ are predefined as described in Sect. 3.1. For the rest of this section we work on the graph $G_n/(\{\odot \}\cup \Gamma _n \cup \Gamma _x)$. Recall that

30 $$\begin{aligned}{} & {} r_\ell = \frac{r}{2^\ell }, \quad \varepsilon _\ell = \frac{\varepsilon }{4^\ell }, \quad k_\ell = \varepsilon _\ell ^{1/2}r_{\ell }, \quad |A_j| \le 2^{13}D^{3}e\varepsilon _{\ell }^{1/3}r_{\ell }, \nonumber \\{} & {} \quad \textrm{Cap}_{k_\ell }(A_j, \Gamma _n \cup \Gamma _x) \ge \frac{2^{11}D^{3}e \varepsilon _{\ell }r_{\ell }^2}{n}. \end{aligned}$$

When we talk about capacity and relative capacity in this section, we are always referring to these quantities on the *original graph* $G_n$.

Recall also that, for each $j \le (2^{13}D^{3}e)^{-1} \varepsilon _{\ell }^{-\frac{1}{3}}$, we let $I_j(k_{\ell })$ be the set of vertices connected to the contracted vertex in ${{\,\textrm{UST}\,}}(G_n/(\{\odot \} \cup \Gamma _n \cup \Gamma _x)$ by a path of length at most $k_{\ell }$, such that the last edge on this path has an endpoint in $A_j$. This also includes vertices originally in $A_j$ before the contraction. Since $\ell$ is fixed for this section, we also set $X_j = |I_j(k_{\ell })|$.

### Claim 5.1

Assume that $\Gamma _x$ and $\Gamma _n$ satisfy (16) (and therefore (30) holds). Fix a scale $\ell$ and consider the graph $G_n / (\Gamma _n\cup \Gamma _x \cup \{\odot \})$ as described above. Then, for every $j\in \{1,\ldots , (2^{13}D^{3}e)^{-1} \varepsilon _{\ell }^{-\frac{1}{3}}\}$ we have

$$\begin{aligned} {\mathbb {E}}[X_j] \ge \frac{n}{2De}\cdot \textrm{Cap}_{k_\ell }(A_j, \Gamma _n \cup \Gamma _x) \ge 2^{10}D^{2}\varepsilon _{\ell }r_{\ell }^2. \end{aligned}$$

### Proof

By Wilson’s algorithm, for every $v\in G_n$, we have that $v\in I_j(k_{\ell })$ if a random walk starting at *v* hits $\Gamma _n \cup \Gamma _x \cup \{\odot \}$ at $A_j$ and its loop erasure is of length at most $k_{\ell }$. Therefore,

$$\begin{aligned} {\mathbb {P}}(v\in I_j) \ge {\mathbb {P}}_v(\tau _\odot> k_{\ell })\cdot {\mathbb {P}}_v(\tau _{A_j}< k_{\ell }\ \text {and} \ \tau _{A_j} < \tau _{(\Gamma _n \cup \Gamma _x) \setminus A_j} \mid \tau _\odot > k_{\ell }), \end{aligned}$$

where all hitting times refer to hitting times of the lazy random walk. First note that ${\mathbb {P}}(\tau _\odot > k_{\ell }) = \left( 1-\frac{1}{k_{\ell }} \right) ^{k_{\ell }} \ge \frac{1}{2e}$. Then, given $\tau _\odot > k_{\ell }$, the lazy random walk until time $k_{\ell }$ is distributed as a lazy random walk on $G_n / (\Gamma _n \cup \Gamma _x)$. Since $G_n$ is balanced with parameter *D* we get

$$\begin{aligned}&{\mathbb {E}}[X_j] = \sum _{v\in G_n}{\mathbb {P}}(v \in I_j) \\&\quad \ge \frac{n}{2De}\sum _{v\in G_n}\pi (v){\mathbb {P}}_v(\tau _{A_j}< k_{\ell }\ \text {and} \ \tau _{A_j} < \tau _{(\Gamma _n \cup \Gamma _x) \setminus A_j} \text {in } G_n/(\Gamma _n\cup \Gamma _x)) \\&\quad \ge \frac{n}{2De}\cdot \textrm{Cap}_{k_\ell }(A_j, \Gamma _n \cup \Gamma _x) \, , \end{aligned}$$

and we conclude the proof using (30). $\square$

Recall that our goal is to find a lower bound for the probability that $\sum _{i=1}^{j+1} X_i$ is large given that $\sum _{i=1}^j X_i$ is small. To this end, let $\Phi _j$ be the (random) edge-set consisting of all simple paths of length at most $k_{\ell }$ in ${{\,\textrm{UST}\,}}(G_n / (\{\odot \} \cup \Gamma _n \cup \Gamma _x))$ that end in the contracted vertex through $A_1 \cup \ldots \cup A_j$. Note that $\Phi _j$ determines $\{\sum _{i=1}^j X_i \le 16\varepsilon _{\ell }r_{\ell }^2\}$ and that conditioning on $\Phi _j = \varphi _j$ for some set of edges $\varphi _j$ means precisely that the edges of $\varphi _j$ are in the ${{\,\textrm{UST}\,}}$ (open edges) and all other edges touching a vertex *v* of $\varphi _j$, such that the path in $\varphi _j$ from *v* to $A_1 \cup \ldots \cup A_j$ is of length at most $k_{\ell }-1$, must not belong to the ${{\,\textrm{UST}\,}}$ (closed edges). These open and closed edges determine $\Phi _j$. Thus, to condition on $\Phi _j = \varphi _j$, we erase the closed edges and contract all the open edges to a single vertex which coincides with $\Gamma _n \cup \Gamma _x \cup \{\odot \}$, and call the remaining graph $G_n(\varphi _j)$. By the spatial Markov property of the ${{\,\textrm{UST}\,}}$ [9, Proposition 4.2] we have that ${{\,\textrm{UST}\,}}(G_n(\varphi _j))$ together with $\varphi _j$ is distributed precisely as ${{\,\textrm{UST}\,}}(G_n / (\{\odot \} \cup \Gamma _n \cup \Gamma _x))$ conditioned on $\Phi _j = \varphi _j$. Note that the event $\{\sum _{i=1}^j X_i \le 16\varepsilon _{\ell }r_{\ell }^2\}$ occurs if and only if $|V(\varphi _j)|\le 16\varepsilon _{\ell }r_{\ell }^2$ where $V(\varphi _j)$ are the vertices touched by $\varphi _j$.

The sun is useful in this section (specifically in Claim 5.2 and Lemma 5.3) as it will help us to upper bound hitting probabilities on the conditioned graph $G_n(\varphi _j)$, and in particular ensures that we only need to consider random walks that run for time of order $k_{\ell }$.

### Claim 5.2

Let $\varphi _j\subset E(G_n)$ be such that ${\mathbb {P}}\left( \Phi _j = \varphi _j\right) >0$ and $|V(\varphi _j)| \le 16\varepsilon _{\ell }r_{\ell }^2$. Let $\gamma$ be a simple path in $G_n(\varphi _j)$ that ends at the contracted vertex. Let $(Y_t)_{t \ge 0}$ denote a lazy random walk on $G_n(\varphi _j)$ started from a uniform vertex *U* of the original graph $G_n$ and killed upon hitting the contracted vertex of $G_n(\varphi _j)/ \gamma$, that is, upon hitting the vertex corresponding to the contracted edges $\{\odot \} \cup \Gamma _n \cup \Gamma _x \cup \varphi _j\cup \gamma$. Denote by $V(\Gamma _n \cup \Gamma _x \cup \varphi _j\cup \gamma )$ the set of vertices of $G_n$ touched by the edges in $\Gamma _n \cup \Gamma _x \cup \varphi _j\cup \gamma$ and let $M \subset V(\Gamma _n \cup \Gamma _x \cup \varphi _j\cup \gamma )$ be a fixed subset of vertices of $G_n$. Then

$$\begin{aligned} {\mathbb {P}}\left( Y \,\,\textrm{ hits }\,\, M\right) \le \frac{64D\varepsilon _{\ell }r_{\ell }^2}{n} + \frac{4D^2|M|k_{\ell }}{n} \, . \end{aligned}$$

(Recall here that to “hit *M*" means to hit the contracted vertex via an edge that originally led to *M*.)

### Proof

Let $\Delta = \max \deg (G_n)$, i.e. the maximal degree in the original graph $G_n$, and let

$$\begin{aligned} V^{\text {bad}}= \left\{ v \in V(G_n) \setminus V(\Gamma _n \cup \Gamma _x \cup \varphi _j\cup \gamma ): \deg _{G_n(\varphi _j)} (v) \le \frac{\deg _{G_n}\left( v\right) }{2}\right\} \,. \end{aligned}$$

In other words, $V^{\text {bad}}$ is the set of all vertices of $G_n$ that are not in the contracted vertex of $G_n(\varphi _j)$ that are adjacent to many closed edges. Since $|V(\varphi _j)|\le 16\varepsilon _{\ell }r_{\ell }^2$, the number of closed edges is no more than $16 \Delta \varepsilon _{\ell }r_{\ell }^2$. Hence the number of vertices touching a closed edge is at most $32 \Delta \varepsilon _{\ell }r_{\ell }^2$ and each vertex in $V^{\text {bad}}$ contributes at least $\Delta /2D$ to this count, so $|V^{\text {bad}}|\le 64D\varepsilon _{\ell }r_{\ell }^2$.

Recall that, when we originally added the sun to $G_n / (\Gamma _n \cup \Gamma _x)$, we chose the weights so that the probability that a lazy random walk on $G_n / (\Gamma _n \cup \Gamma _x)$ would jump to the sun at the next step is always $\frac{1}{k_{\ell }}$. In the graph $G_n(\varphi _j)$, we have now contracted some edges and closed some other edges. For any $x \in G_n(\varphi _j)$, these operations can only increase the probability that *Y* will jump directly to the sun from the vertex *x*. Therefore, by coupling, we can separate the sun and its incident edges, and obtain an upper bound for ${\mathbb {P}}\left( Y \,\,\textrm{ hits }\,\, M\right)$ by instead bounding the same probability for a lazy random walk on $(G_n(\varphi _j)/\gamma ) {\setminus } \{ \odot \}$ with an independent Geo$(\frac{1}{k_{\ell }})$ killing time. We denote this second lazy random walk by $Y'$.

To control capacity on $(G_n(\varphi _j)/\gamma ) \setminus \{ \odot \}$ we will need to work with the stationary measure on $(G_n(\varphi _j)/\gamma ) {\setminus } \{ \odot \}$, which we denote by $\pi '$ (the bound on $|V^{\text {bad}}|$ above will then help us to compare $\pi '$ with the uniform measure). We define $\pi '$ on all of $G_n$ by remembering the edges from before the contraction. In particular, this means that for $u \in G_n$, we have

$$\begin{aligned} \pi ' (u) = \frac{\deg _{G_n}(u) - N^{\textrm{cl}}(u)}{\sum _{v \in G_n}(\deg _{G_n}(v) - N^{\textrm{cl}}(v))}, \end{aligned}$$

where $N^{\textrm{cl}}(v)$ denotes the number of closed edges incident to *v* in $G_n(\varphi _j)$.

We now observe the following. If $u \in G_n \setminus V^{\text {bad}}$, then

$$\begin{aligned} \pi '(u) \ge \frac{\deg _{G_n}\left( u\right) /2}{\sum _{v \in G_n} \deg _{G_n}(v)} \ge \frac{\pi (u)}{2}. \end{aligned}$$

Also, for every $u \in G_n$, provided that $c<\sqrt{1/64D}$ and $\varepsilon <1$ and using the fact that $\sum _{v\in G_n} N^{\textrm{cl}}(v) \le 32\Delta \varepsilon _{\ell }r_{\ell }^2$, we have that

$$\begin{aligned} \sum _{v\in G_n} \left( \deg _{G_n}(v) - N^{\textrm{cl}}(v)\right)&geq{\sum _{v\in G_n} \deg _{G_n}(v) - 32 \Delta \varepsilon _{\ell }r_{\ell }^2} \ge \sum _{v\in G_n} \deg _{G_n}(v) \\&\quad - \frac{\Delta n}{2D} \ge \frac{1}{2}\sum _{v\in G_n} \deg _{G_n}(v). \end{aligned}$$

Therefore,

$$\begin{aligned} \pi '(u) \le \frac{\deg _{G_n}(u)}{\sum _{v\in G_n} \left( \deg _{G_n}(v) - N^{\textrm{cl}}(v)\right) } \le 2\pi \left( u\right) . \end{aligned}$$

In what follows, these two observations mean that we will be able to switch between $\pi '$ and $\pi$ and vice versa provided we multiply by 2. In particular, we can write

$$\begin{aligned}&{\mathbb {P}}_{U} \left( Y' \text { hits } M\right) \le {\mathbb {P}}\left( U \in V^{\text {bad}}\right) + 2D{\mathbb {P}}_{\pi '}\left( Y' \text { hits } M \right) \\&\quad \le \frac{64D\varepsilon _{\ell }r_{\ell }^2}{n} + 2D\sum _{t=0}^{\infty } {\mathbb {P}}_{\pi '} \left( Y_t' \in M\right) \\&\quad = \frac{64D\varepsilon _{\ell }r_{\ell }^2}{n} + 2D\sum _{t=0}^{\infty } \pi ' (M) {\mathbb {P}}\left( \textsf {Geo}\left( \frac{1}{k_{\ell }}\right) \ge t \right) \\&\quad \le \frac{64D\varepsilon _{\ell }r_{\ell }^2}{n} + \frac{4D^2|M|}{n} \sum _{t=0}^\infty \left( 1 - \frac{1}{k_{\ell }}\right) ^t = \frac{64D\varepsilon _{\ell }r_{\ell }^2}{n} + \frac{4D^2|M|k_{\ell }}{n}. \end{aligned}$$

$$\square $$

We will use Claim 5.2 to prove the following upper bounds.

### Lemma 5.3

Let $\varphi _j\subset E(G_n)$ be such that ${\mathbb {P}}\left( \Phi _j = \varphi _j\right) >0$ and $|V(\varphi _j)| \le 16\varepsilon _{\ell }r_{\ell }^2$. Then

(i) $${\mathbb {E}}\left[ X_{j+1} \mid \Phi _j = \varphi _j\right] \le 5D^5 \cdot 2^{13} \cdot e \cdot \varepsilon _{\ell }^{5/6}r_{\ell }^2$$.
(ii) $${{\,\textrm{Var}\,}}\left( X_{j+1} \mid \Phi _j = \varphi _j\right) \le 68D^2\varepsilon _{\ell }r_{\ell }^2 {\mathbb {E}}\left[ X_{j+1}\mid \Phi _j = \varphi _j\right] $$,

### Proof

We condition on $\Phi _j = \varphi _j$ throughout this proof so our probability space is that of ${{\,\textrm{UST}\,}}(G_n(\varphi _j))$. To prove (i) we condition on $\Phi _j = \varphi _j$ and take any $v \in G_n(\varphi _j)\setminus \{\odot \}$. By Wilson’s algorithm on the graph $G_n(\varphi _j)$, we have that ${\mathbb {P}}\left( v \in A_{j+1} \mid \Phi _j = \varphi _j\right)$ is upper bounded by the probability that a lazy random walk started at *v* hits $A_{j+1}$ before it hits the sun. If $(Y_t)_{t \ge 0}$ is such a random walk starting from a uniform vertex of $G_n$, by Claim 5.2 and (30) we have that

$$\begin{aligned} {\mathbb {E}}\left[ X_{j+1} \mid \Phi _j = \varphi _j\right]&\le n{\mathbb {P}}\left( Y_t \text { hits } A_{j+1}\right) \le 64D\varepsilon _{\ell }r_{\ell }^2 + 4D^2|A_{j+1}|k_{\ell }\le 5D^2|A_{j+1}|k_{\ell }\\&\le 5D^5 \cdot 2^{13} \cdot e \cdot \varepsilon _{\ell }^{5/6}r_{\ell }^2 \, , \end{aligned}$$

where we also used the lower and upper bounds on $|A_{j+1}|$ in (30).

To ease notation in the proof of (ii) we write ${\mathbb {P}}(\cdot ), {\mathbb {E}}\left[ \cdot \right]$ and $\text {Var}(\cdot )$ for ${\mathbb {P}}\left( \cdot \mid \Phi _j = \varphi _j\right)$ and the corresponding expectation and variance. We have

31 $$\begin{aligned} {{\,\textrm{Var}\,}}(X_{j+1})&= \sum _{u,v \in G_n} {\mathbb {P}}(u,v \in I_{j+1}) - {\mathbb {P}}(u\in I_{j+1}){\mathbb {P}}(v\in I_{j+1}).\\&= \sum _v \sum _u \Big [{\mathbb {P}}\left( u \in I_{j+1} \mid v \in I_{j+1}\right) - {\mathbb {P}}(u\in I_{j+1})\Big ]{\mathbb {P}}(v\in I_{j+1}). \nonumber \end{aligned}$$

Fix some *v*, and rewrite the inner sum as

$$\begin{aligned} n\big [ {\mathbb {P}}(U\in I_{j+1} \mid v\in I_{j+1}) - {\mathbb {P}}(U \in I_{j+1})\big ], \end{aligned}$$

where *U* is a vertex chosen uniformly from $G_n$. We decompose the event $v\in I_{j+1}$ according to $\gamma _v$, the path from *v* to $A_{j+1}$ in $G_n(\varphi _j)$ which is of length at most $k_{\ell }$ and obtain that

$$\begin{aligned}&{\mathbb {P}}(U\in I_{j+1} \mid v\in I_{j+1}) - {\mathbb {P}}(U \in I_{j+1}) = \sum _{\gamma _v}\\ {}&{\mathbb {P}}(\gamma _v \subseteq {{\,\textrm{UST}\,}}(G_n(\varphi _j)) \mid v\in I_{j+1})\left[ {\mathbb {P}}(U\in I_{j+1} \mid \gamma _v \subseteq {{\,\textrm{UST}\,}}(G_n(\varphi _j))) - {\mathbb {P}}(U\in I_{j+1})\right] . \end{aligned}$$

To compare ${\mathbb {P}}(U\in I_{j+1} \mid \gamma _v\subseteq {{\,\textrm{UST}\,}}(G_n(\varphi _j)))$ and ${\mathbb {P}}(U\in I_{j+1})$ we note again from the spatial Markov property [9, Proposition 4.2] that the rest of ${{\,\textrm{UST}\,}}(G_n(\varphi _j))$ given $\gamma _v\subseteq {{\,\textrm{UST}\,}}(G_n(\varphi _j))$ is the ${{\,\textrm{UST}\,}}$ on the graph obtained from $G_n(\varphi _j)$ by contracting $\gamma _v$. By coupling Wilson’s Algorithm running on each of the two graphs $G_n(\varphi _j)$ and $G_n(\varphi _j)/\gamma _v$, the difference between the two quantities can be upper bounded by the probability that a random walk starting from a uniform vertex of $G_n$ hits $\gamma _v$ before it hits the new sun $\odot$. By Claim 5.2, this is bounded by $\frac{64D\varepsilon _{\ell }r_{\ell }^2}{n} + \frac{4D^2k_{\ell }^2}{n}$ uniformly for all $\gamma _v$ with $|\gamma _v| \le k_{\ell }$. As $\sum _{\gamma _v}{\mathbb {P}}(\gamma _v \subseteq {{\,\textrm{UST}\,}}(G_n(\varphi _j)) \mid v\in I_{j+1})$ sums to 1 we obtain that

$$\begin{aligned} n\left( {\mathbb {P}}(U\in I_{j+1} \mid v\in I_{j+1}) - {\mathbb {P}}(U \in I_{j+1})\right) \le 64D\varepsilon _{\ell }r_{\ell }^2 + 4D^2k_{\ell }^2. \end{aligned}$$

Plugging this into (31) and using (30) we obtain

$$\begin{aligned} {{\,\textrm{Var}\,}}(X_{j+1})\le & {} \sum _v (64D\varepsilon _{\ell }r_{\ell }^2 + 4D^2k_{\ell }^2) {\mathbb {P}}(v\in I_{j+1}) \le (64D\varepsilon _{\ell }r_{\ell }^2 + 4D^2k_{\ell }^2) {\mathbb {E}}[X_{j+1}] \\= & {} 68D^2\varepsilon _{\ell }r_{\ell }^2 {\mathbb {E}}[X_{j+1}]. \end{aligned}$$

$$\square $$

Recall that $B^{\odot }_j= \{ \sum _{i=1}^j X_i \le 16\varepsilon _{\ell }r_{\ell }^2\}$, and $\Phi _j$ is the random edge-set induced by $\cup _{i=1}^j I_i (k_{\ell })$. Under $B^{\odot }_j$, we have no information about the structure of $\Phi _j$, other than that $|\Phi _j| \le 16\varepsilon _{\ell }r_{\ell }^2$ (and this was important for the factorization in the proof of Corollary 3.8). However, in order to prove Lemma 3.7, we will need the following lower bound.

### Lemma 5.4

It holds that

$$\begin{aligned} {\mathbb {P}}\left( \Phi _j \in \{\varphi _j: {\mathbb {E}}[X_{j+1} | \Phi _j = \varphi _j] \ge 2^9D^{2}\varepsilon _{\ell }r_{\ell }^2 \} \mid B^{\odot }_j\right) \ge \frac{\varepsilon _{\ell }^{1/6}}{80D^3e}. \end{aligned}$$

### Proof

Recall that we are working on the graph $G_n/(\Gamma _n\cup \Gamma _x \cup \{\odot \})$. Suppose that $\sum _{i=1}^j X_i \le 16\varepsilon _{\ell }r_{\ell }^2$, and note that this event can be written as the disjoint union of all possible $\varphi _j$ such that ${\mathbb {P}}(\Phi _j = \varphi _j)>0$ and $|V(\varphi _j)|\le 16\varepsilon _{\ell }r_{\ell }^2$. When conditioning on $\Phi _j = \varphi _j$ for some $\varphi _j$ we work on the graph $G_n(\varphi _j)$, as defined above Claim 5.2. Note that by Lemma 5.3, we have that for every $\varphi _j$ with $|V(\varphi _j)| \le 16\varepsilon _{\ell }r_{\ell }^2$ and ${\mathbb {P}}\left( \Phi _j = \varphi _j\right) >0$ that

$$\begin{aligned} {\mathbb {E}}\left[ X_{j+1} | \Phi _j = \varphi _j\right] \le 5D^5 \cdot 2^{13} e \varepsilon _{\ell }^{5/6}r_{\ell }^2. \end{aligned}$$

Furthermore, by Claim 2.12 and Claim 5.1 we have that

$$\begin{aligned} {\mathbb {E}}\left[ X_{j+1} \big \vert \sum _{i=1}^j X_i \le 16\varepsilon _{\ell }r_{\ell }^2 \right] \ge {\mathbb {E}}\left[ X_{j+1}\right] \ge 2^{10}D^{2}\varepsilon _{\ell }r_{\ell }^2. \end{aligned}$$

Write ${\mathbb {E}}'$ and ${\mathbb {P}}'$ for the expectation and probability operators ${\mathbb {P}}(\cdot \mid B^{\odot }_j)$ and ${\mathbb {E}}\left[ \cdot \mid B^{\odot }_j\right]$ on $G_n / (\{\odot \} \cup \Gamma _n \cup \Gamma _x)$. We have that

$$\begin{aligned} {\mathbb {E}}'\left[ X_{j+1} \big \vert \Phi _j \right]&\le 5 D^{5}\cdot 2^{13} \cdot e\cdot \varepsilon _{\ell }^{5/6}r_{\ell }^2\quad \text {a.s.},\\ {\mathbb {E}}'\left[ {\mathbb {E}}'\left[ X_{j+1} \mid \Phi _j\right] \right]&= {\mathbb {E}}'[X_{j+1}] \ge 2^{10}D^{2}\varepsilon _{\ell }r_{\ell }^2. \end{aligned}$$

Therefore

$$\begin{aligned}{} & {} 2^{10}D^{2}\varepsilon _{\ell }r_{\ell }^2 \le {\mathbb {E}}'[X_{j+1}] \\{} & {} \quad \le 2^9D^{2}\varepsilon _{\ell }r_{\ell }^2 + {\mathbb {P}}'[{\mathbb {E}}'[X_{j+1} \mid \Phi _j] \ge 2^9D^{2}\varepsilon _{\ell }r_{\ell }^2] \cdot 5 D^5 \cdot 2^{13} \cdot e \varepsilon _{\ell }^{5/6}r_{\ell }^2. \end{aligned}$$

Rearranging, we deduce that

$$\begin{aligned} {\mathbb {P}}'\left( {\mathbb {E}}'[X_{j+1} \mid \Phi _j] \ge 2^9D^{2}\varepsilon _{\ell }r_{\ell }^2\right) \ge \frac{\varepsilon _{\ell }^{1/6}}{80D^3e}, \end{aligned}$$

as required. $$\square $$

### Lemma 5.5

Suppose $\varphi _j$ is such that ${\mathbb {P}}\left( \Phi _j = \varphi _j\right) >0$ and ${\mathbb {E}}\left[ X_{j+1} \mid \Phi _j = \varphi _j\right] \ge 2^9D^{2}\varepsilon _{\ell }r_{\ell }^2$. Then

$$\begin{aligned} {\mathbb {P}}\left( X_{j+1} \le 16\varepsilon _{\ell }r_{\ell }^2 \mid \Phi _j = \varphi _j\right) \le \frac{1}{2}. \end{aligned}$$

### Proof

The result is a straightforward application of Chebyshev’s inequality, similarly to [19, Lemma 6.13]. First note that it follows from Lemma 5.3(ii) that

$$\begin{aligned} \text {Var}\left( X_{j+1} \mid \Phi _j = \varphi _j\right) \le 68D^2\varepsilon _{\ell }r_{\ell }^2{\mathbb {E}}\left[ X_{j+1} \mid \Phi _j = \varphi _j\right] \le \frac{68}{2^{9}}{\mathbb {E}}\left[ X_{j+1} \mid \Phi _j = \varphi _j\right] ^2, \end{aligned}$$

where in the last inequality we used that ${\mathbb {E}}\left[ X_{j+1} \big \vert \Phi _j = \varphi _j\right] \ge 2^{9}D^{2}\varepsilon _{\ell }r_{\ell }^2$ by assumption. Using this again we therefore deduce that

$$\begin{aligned} {\mathbb {P}}\left( X_{j+1} \le 16\varepsilon _{\ell }r_{\ell }^2 \big \vert \Phi _j = \varphi _j\right)&\le {\mathbb {P}}\left( X_{j+1} \le \frac{1}{2^{5}D^{2}}{\mathbb {E}}\left[ X_{j+1} \big \vert \Phi _j = \varphi _j\right] \big \vert \Phi _j = \varphi _j\right) \\&\le \frac{2 \text {Var}\left( X_{j+1} \big \vert \Phi _j = \varphi _j\right) }{{\mathbb {E}}\left[ X_{j+1} \big \vert \Phi _j = \varphi _j\right] ^2} \le \frac{1}{2}. \end{aligned}$$

$$\square $$

### Proof of Lemma 3.7

By Lemma 5.4, given that $\sum _{i=1}^j X_i \le 16\varepsilon _{\ell }r_{\ell }^2$, we get with probability at least $\varepsilon _{\ell }^{1/6}/(80D^{3}e)$ that $\varphi _j$ satisfies

$$\begin{aligned} {\mathbb {E}}[X_{j+1} \mid \Phi _j=\varphi _j] \ge 2^9D^{2}\varepsilon _{\ell }r_{\ell }^2. \end{aligned}$$

For every such $\varphi _j$, by Lemma 5.5, we get that given $\Phi _j = \varphi _j$, we have that $X_{j+1} \ge 16\varepsilon _{\ell }r_{\ell }^2$ with probability at least 1/2. We conclude that

$$\begin{aligned} {\mathbb {P}}\left( B^{\odot }_{j+1}\big \vert B^{\odot }_j, (\Gamma _n \cup \Gamma _x), \textrm{Cap}_{k_\ell }(\Gamma _x^{5r_{\ell }/6}, \Gamma _n \cup \Gamma _x ) \ge \frac{ r_{\ell }k_{\ell }\varepsilon _{\ell }^{\frac{1}{6}}}{n} \right) \le 1 - \frac{\varepsilon _{\ell }^{1/6}}{160D^{3}e}, \end{aligned}$$

as required. $$\square $$

## A Criterion for GHP Convergence

### GP convergence

We first aim to address the convergence provided in Theorem 3.1. Recall our definitions and notation from Sect. 1.1 (in fact this section can be seen as a direct continuation of Sect. 1.1).

#### Definition 6.1

Let $(X,d,\mu )$ and $(X',d',\mu ')$ be elements of ${\mathbb {X}}_c$. The **Gromov–Prohorov (GP) pseudo-distance** between $(X,d,\mu )$ and $(X',d',\mu ')$ is defined as

$$\begin{aligned} d_{\textrm{GP}}((X,d,\mu ),(X',d',\mu ')) = \inf \left\{ d_P(\phi _* \mu , \phi _*' \mu ') \right\} , \end{aligned}$$

where the infimum is taken over all isometric embeddings $\phi : X \rightarrow F$, $\phi ': X' \rightarrow F$ into some common metric space *F*.

It is important to highlight that when the measures do not have full support, two mm-spaces can be equivalent in the GP sense even though the GHP distance between them can be positive. Thus $d_{\textrm{GP}}$ is a metric on the space ${\mathbb {X}}_c^{\textrm{GP}}$, which is the space ${\mathbb {X}}_c$ in which we identify all mm-spaces with $\textrm{GP}$ distance 0. There is a useful equivalent definition of convergence of mm-spaces with respect to the $\textrm{GP}$ distance. Given an mm-space $(X,d,\mu )$ and a fixed $m \in {\mathbb {N}}$ we define a measure $\nu _m((X,d,\mu ))$ on ${\mathbb {R}}^{m \atopwithdelims ()2}$ to be the law of the ${m \atopwithdelims ()2}$ pairwise distances between *m* i.i.d. points drawn according to $\mu$.

#### Theorem 6.2

(Theorem 5 in [16])**.** Let $(X_n,d_n,\mu _n)$ and $(X,d,\mu )$ be elements of ${\mathbb {X}}_c^{\textrm{GP}}$. Then

$$\begin{aligned} d_{\textrm{GP}}( (X_n,d_n,\mu _n), (X,d,\mu )) \longrightarrow 0 \,, \end{aligned}$$

if and only if for any $m\in {\mathbb {N}}$

$$\begin{aligned} \nu _m((X_n,d_n, \mu _n)) \Rightarrow \nu _m((X,d,\mu )) \,, \end{aligned}$$

where $\Rightarrow$ denotes standard weak convergence of measures on ${\mathbb {R}}^{m \atopwithdelims ()2}$.

This is still not quite the setting of this paper since ${{\,\textrm{UST}\,}}$s are *random* mm-spaces. Thus let ${\mathcal {M}}_1({\mathbb {X}}_c^{{\textrm{GP}}})$ denote the space of probability measures on ${\mathbb {X}}_c^{\textrm{GP}}$. Each element ${\mathbb {P}}\in {\mathcal {M}}_1({\mathbb {X}}_c^{\textrm{GP}})$ therefore defines random measures $\left( \nu _m\right) _{m\ge 2}$ and we additionally have annealed measures on $${\mathbb {R}}^{\left( {\begin{array}{c}m\\ 2\end{array}}\right) }$$, given by

$$\begin{aligned} {\tilde{\nu }}_m({\mathbb {P}}):= \int _{{\mathbb {X}}_c^{\textrm{GP}}} \nu _m((X,d,\mu ))d{\mathbb {P}}\end{aligned}$$

for each integer $m\ge 2$. It is often more straightforward to prove deterministic weak convergence of the measures ${\tilde{\nu }}_m$ for each $m\ge 2$, rather than distributional weak convergence of the random measures $\nu _m$ for each *m*. For example, the conclusion of Theorem 3.1 can be restated as

32 $$\begin{aligned} {\tilde{\nu }}_m \left( \left( {{\,\textrm{UST}\,}}(G_n), d_n/(\beta _n \sqrt{n}), \mu _n\right) \right) \Rightarrow {\tilde{\nu }}_m({{\,\textrm{CRT}\,}}) , \end{aligned}$$

for any fixed $m\ge 2$. However, it may not be immediately clear that this implies that the ${{\,\textrm{UST}\,}}$s converge to the ${{\,\textrm{CRT}\,}}$ in distribution with respect to the topology of $({\mathbb {X}}_c^{\textrm{GP}},d_{\textrm{GP}})$; we address this in the next lemma.

#### Lemma 6.3

Suppose that $(G_n)_{n \ge 1}$ is a sequence of graphs satisfying Assumption 1.4. Let $d_n$ denote the graph distance on ${{\,\textrm{UST}\,}}(G_n)$ and $\mu _n$ the uniform probability measure on its vertices. Then there exists a sequence $(\beta _n)_n$ satisfying $0<\inf _n \beta _n \le \sup _n \beta _n < \infty$ such that $({{\,\textrm{UST}\,}}(G_n),\frac{1}{\beta _n \sqrt{n}}d_n, \mu _n)$ converges in distribution to the ${{\,\textrm{CRT}\,}}$ with respect the topology of $({\mathbb {X}}_c^{\textrm{GP}},d_{\textrm{GP}})$.

#### Proof

We appeal to [16, Corollary 3.1] and verify conditions (i) and (ii) there. Condition (ii) is precisely (32). To verify condition (i) we use [16, Theorem 3] (and recall that by Prohorov’s Theorem the relative compactness of the measures is equivalent to their tightness) and verify conditions (i) and (ii) there (see also Proposition 8.1 in [16]). Condition (i) is just saying that ${\tilde{\nu }}_2$ is a tight sequence of measures on ${\mathbb {R}}$, which follows from (32). Lastly, Theorem 3.2 directly implies condition (ii) [16, Theorem 3]. $\square$

We remark that the use of Theorem 3.2 in the last line of the proof above is an overkill and it is not too difficult to verify condition (ii) of [16, Theorem 3] directly using (32).

### GHP convergence and the lower mass bound

The key to strengthening the $\textrm{GP}$ convergence of [34], as stated in Lemma 6.3, to $\textrm{GHP}$ convergence is the lower mass bound criterion of [7]. In [7, Theorem 6.1] it is shown that $\textrm{GP}$ convergence of deterministic mm-spaces together with this criterion is equivalent to $\textrm{GHP}$ convergence. In this paper we require an extension to the setting of random mm-spaces (i.e., measures on mm-spaces); it is not hard to obtain this using the ideas of [7] and we provide it here (Proposition 6.5).

As in [7, Section 3], given $c > 0$ and an mm-space $(X,d,\mu )$ we define

$$\begin{aligned} m_c((X,d,\mu ))&= \inf _{x \in X}\{\mu (B(x, c))\} \, . \end{aligned}$$

We begin with a short claim about deterministic mm-spaces.

#### Claim 6.4

Let $(X_n,d_n,\mu _n)$ be a sequence of mm-spaces that is $\textrm{GP}$-convergent to $(X,d,\mu )$, i.e.,

$$\begin{aligned} d_{\textrm{GP}}( (X_n,d_n,\mu _n), (X,d,\mu )) \rightarrow 0 \,. \end{aligned}$$

Suppose further that for every $c>0$ we have

$$\begin{aligned} \inf _n m_c((X_n,d_n,\mu _n)) > 0 \,. \end{aligned}$$

Then, for every $\varepsilon >0$,

$$\begin{aligned} \inf _{x\in \textrm{supp}(\mu )} \mu (B(x,\varepsilon )) \ge \liminf _{n \rightarrow \infty }\inf _{x\in X_n} \mu _n\left( B(x,\varepsilon /2)\right) > 0. \end{aligned}$$

#### Proof

Fix some $x\in \textrm{supp}(\mu )$ and $\varepsilon >0$. Then $\mu (B(x,\varepsilon /4)) \ge b$ for some $b=b(x,\varepsilon )>0$. Put $\delta = \min \{b/2,\varepsilon /12\}$. By the $\textrm{GP}$ convergence there exists $N\in {\mathbb {N}}$ such that for every $n\ge N$ there are isometric embeddings taking $X_n$ and *X* to a common metric space $(E,d_n')$ such that the Prohorov distance between the pushforwards of their measures is smaller than $\delta$. Therefore we may assume that $X_n$ and *X* are both subsets of some common metric space. We abuse notation and write $\mu$ and $\mu _n$ in place of their respective pushforward measures. Since the $\textrm{GP}$ distance is at most $\delta$ we get that

$$\begin{aligned} b\le \mu (B(x,\varepsilon /4)) \le \mu _n(B(x,\varepsilon /4+\delta ))+\delta . \end{aligned}$$

for $n\ge N$. Hence by our choice of $\delta$ we get

$$\begin{aligned} \mu _n(B(x,\varepsilon /3)) > 0. \end{aligned}$$

Therefore, we can find some $y_n\in X_n$ such that $d_n'(x,y_n) < \varepsilon /3$. Also, for any $\delta '\in (0,\varepsilon /6)$, we can find $N_2 \in {\mathbb {N}}$ such that for $n\ge N_2$ we have

$$\begin{aligned} \inf _{y\in X_n} \mu _n(B(y,\varepsilon /2)) \le \mu _n(B(y_n,\varepsilon /2)) \le \mu (B(y_n,\varepsilon /2+\delta ')) + \delta ' \le \mu (B(x,\varepsilon )) + \delta '. \end{aligned}$$

Hence, taking the $\liminf$ on the left hand side and then taking $\delta ' \rightarrow 0$ we obtain that for all $x\in X$

$$\begin{aligned} \liminf _{n \rightarrow \infty }\inf _{y\in X_n} \mu _n(B(y,\varepsilon /2)) \le \mu (B(x,\varepsilon )), \end{aligned}$$

and the claim follows by taking the infimum over $x\in X$. $\square$

We now state and prove the main goal of this section; as we state immediately afterwards, it readily shows that Theorem 3.2 implies Theorem 1.6.

#### Theorem 6.5

Let $((X_n,d_n,\mu _n))_{n\ge 1}, (X,d,\mu )$ be random mm-spaces in ${\mathbb {X}}_c$ and suppose that

(i) $(X_n, d_n, \mu _n) \overset{(d)}{\longrightarrow } (X,d,\mu )$ with respect to the $\textrm{GP}$ topology.
(ii) For every $c > 0$, the sequence $\left( m_c((X_n,d_n,\mu _n))^{-1}\right) _{n \ge 1}$ is tight.

Then $(X_n, d_n, \mu _n) \overset{(d)}{\rightarrow }\ (\textrm{supp}(\mu ),d,\mu )$ with respect to the $\textrm{GHP}$ topology.

#### Proof

The metric space $({\mathbb {X}}_c,d_{\textrm{GP}})$ is separable (see [7, Figure 1]), hence by the Skorohod Representation theorem, there exists a probability space on which the convergence in (*i*) holds almost surely. We will henceforth work on this probability space, and may therefore assume that $(X_n)_{n\ge 1}$ and *X* are embedded in a common metric space where $d_P(X,X_n) \rightarrow 0$ almost surely. We will show that on this probability space, we have that $(X_n, d_n, \mu _n) \longrightarrow (\textrm{supp}(\mu ),d,\mu )$ in *probability* with respect to the $\textrm{GHP}$ topology, giving the required assertion.

Let $\varepsilon , \varepsilon _2 >0$. By (*ii*), we have that there exists some $c_1>0$ and $N_1\in {\mathbb {N}}$ such that for every $n\ge N_1$ we have

$$\begin{aligned} {\mathbb {P}}\left( \inf _{x\in X_n} \mu _n(B_{d_n}(x,\varepsilon /2)) \le c_1\right) \le \varepsilon _2. \end{aligned}$$

Hence by Fatou’s lemma

$$\begin{aligned} {\mathbb {P}}\left( \limsup _{n} \left\{ \inf _{x\in X_n} \mu _n(B_{d_n}(x,\varepsilon /2)) > c_1 \right\} \right) \ge 1-\varepsilon _2. \end{aligned}$$

Meaning, with probability larger than $1-\varepsilon _2$, we can find a (random) subsequence $n_k$ such that for every $k\in {\mathbb {N}}$ we have that

$$\begin{aligned} m_{\varepsilon /2}((X_{n_k},d_{n_k},\mu _{n_k})) = \inf _{x\in X_{n_k}} \mu _{n_k}(B_{d_{n_k}}(x,\varepsilon /2)) > c_1. \end{aligned}$$

Hence by Claim 6.4, on this event we have that $\inf _{x\in \textrm{supp}(\mu )} \mu (B_d(x,\varepsilon )) \ge c_1$. Next, since almost sure convergence implies convergence in probability, we get by assumption (i) that

$$\begin{aligned} \lim _{n\rightarrow \infty }{\mathbb {P}}\left( d_P\left( (X_n, d_n, \mu _n),(X,d,\mu )\right) > \varepsilon \wedge \frac{c_1}{2} \right) = 0. \end{aligned}$$

Hence we can find $N_2\in {\mathbb {N}}$ such that for every $n\ge \max \{N_1,N_2\}$ with probability at least $1-3\varepsilon _2$ the three events

$$\begin{aligned}{} & {} \inf _{x\in X_n} \mu _n(B_{d_n}(x,\varepsilon )) \ge c_1 \,, \,\, \inf _{x\in \textrm{supp}(\mu )} \mu (B_d(x,\varepsilon )) \ge c_1 \,\,, \,\, d_P \left( (X_n, d_n, \mu _n),(X,d,\mu )\right) \\{} & {} \quad \le \varepsilon \wedge \frac{c_1}{2} \end{aligned}$$

occur. Let $x \in \textrm{supp}(\mu )$. On these events, since the Prohorov distance between $\mu$ and $\mu _n$ is smaller than $\varepsilon \wedge \frac{c_1}{2}$, we have that

$$\begin{aligned} c_1 \le \mu (B_d(x,\varepsilon )) \le \mu _n(B_{d_n}(x,2\varepsilon )) + \frac{c_1}{2}. \end{aligned}$$

Hence

$$\begin{aligned} \mu _n(B_{d_n}(x,2\varepsilon )) > 0. \end{aligned}$$

Thus, $\textrm{supp}(\mu ) \subseteq X_n^{2\varepsilon }$. We use the same argument to obtain that under this event, $X_n \subseteq \textrm{supp}(\mu )^{2\varepsilon }$ and conclude that

$$\begin{aligned} {\mathbb {P}}\left( d_{\textrm{GHP}}\left( (X_n, d_n, \mu _n),(\textrm{supp}(\mu ),d,\mu )\right) > 2\varepsilon \right) \le 3\varepsilon _2. \end{aligned}$$

We therefore get that $(X_n,d_n,\mu _n)$ converges in probability (hence, in distribution) to $(\textrm{supp}(\mu ),d,\mu )$ in the Gromov–Hausdorff–Prohorov topology, as required. $\square$

#### Proof of Theorem 1.6

Lemma 6.3 shows that the ${{\,\textrm{UST}\,}}$ sequence converges in distribution with respect to $d_{\textrm{GP}}$ to the CRT $(X,d,\mu )$ so that condition (i) of Proposition 6.5 holds. Theorem 3.2 verifies that condition (ii) holds, and lastly, it is well known (see [1, Theorem 3]) that $\textrm{supp}(\mu )=X$. The conclusion of Proposition 6.5 thus verifies Theorem 1.6. $\square$

## Comments and Open Questions

Combining with self-similarity of the CRT, Theorem 1.1 can also be used to recover the UST scaling limit in other settings. For instance, Theorem 2 of [3] entails that the branch point between three uniformly chosen points in the CRT splits the CRT into three smaller copies of itself, with masses distributed according to the Dirichlet$(\frac{1}{2}, \frac{1}{2}, \frac{1}{2})$ distribution, and where each copy is independent of the others after rescaling. This together with Theorem 1.1 shows the following.

### Example 7.1

Set $G_n = {\mathbb {Z}}_{\lfloor n^\frac{1}{d}\rfloor }^d$, the torus on (approximately) *n* vertices and $d>4$. Sample a Dirichlet$(\frac{1}{2}, \frac{1}{2}, \frac{1}{2})$ random variable, that is, a uniform triplet $(\Delta _1, \Delta _2, \Delta _3)$ on the 2-simplex. Conditioned on this, let $G_{\lfloor \Delta _1 n \rfloor }$, $G_{\lfloor \Delta _2 n \rfloor }$, and $G_{\lfloor \Delta _3 n \rfloor }$ be disjoint and attach each to an outer vertex of a 3-star. Let $T_n$ be the ${{\,\textrm{UST}\,}}$ on the resulting graph and $\mu _n$ the uniform measure on its vertices. Then $(T_n, \frac{1}{\beta (d) \sqrt{n}} d_{T_n}, \mu _n) \overset{(d)}{\longrightarrow }\ ({\mathcal {T}},d_{{\mathcal {T}}},\mu )$.

Next, building on the corollaries in Sect. 1.3, one can also ask finer questions about the structure of the UST in the mean-field regime. One in particular is the convergence of the height profile.

### Problem 7.2

Take the setup of Theorem 1.6, and set $H_n(r) = \#\{v \in G_n: d_{{\mathcal {T}}_n}(O,v) = r \}$. Does the process $\left( H_n(r\beta _n\sqrt{n})/\sqrt{n}\right) _{r > 0}$ converge to its continuum analogue on the CRT? (That is, the Brownian local time process $(\ell (r))_{r \ge 0}$ defined in [15, Theorem 1.1]).

This does not follow straightforwardly from the GHP convergence of Theorem 1.6 since that only captures the convergence of full balls of diameter $\sqrt{n}$ with volumes of order *n*. (On the other hand, it is straightforward prove convergence of the rescaled volume profile $V_n(r) = \sum _{s \le r} H_n(s)$ from GHP convergence.)

Next, our paper addresses the general mean-field case but leaves the upper critical dimension case of ${\mathbb {Z}}_{n^{1/4}}^4$ open. Here the mixing time is really of order $n^{1/2}$, but it was shown by Schweinsberg [36] that Gromov-weak convergence to the CRT still holds with an additional scaling factor of $(\log n)^{1/6}$. Our proof of the lower mass bound does not immediately transfer to the 4-dimensional setting, leaving this question open.

### Problem 7.3

Let ${\mathcal {T}}_n$ be a uniformly drawn spanning tree of the 4-dimensional torus ${\mathbb {Z}}_n^4$. Denote by $d_{{\mathcal {T}}_n}$ the corresponding graph-distance in ${\mathcal {T}}_n$ and by $\mu _n$ the uniform probability measure on the vertices of ${\mathcal {T}}_n$. Let $\gamma _n$ be the sequence appearing in [36, Theorem 1.1], uniformly bounded away from 0 and infinity. Does the lower mass bound of Proposition 6.5(ii) hold for the sequence $\left( {\mathcal {T}}_n,\frac{d_{{\mathcal {T}}_n}}{\gamma _n n^{2}(\log n)^{1/6}},\mu _n\right) _{n \ge 1}$?

Note that some preliminary estimates on lower volume tails of uniform spanning forests of ${\mathbb {Z}}^4$ were recently obtained by Halberstam and Hutchcroft in [17]. In particular they show that ${\mathbb {P}}\left( |B(0,r)| \le \varepsilon r^2 (\log r)^{-1/3} \right)$ is upper bounded by $O\left( \varepsilon ^{1/5}\right) + O\left( \frac{\log \log r}{(\log r)^{2/3}}\right)$, also using some results of Hutchcroft and Sousi [20] on four dimensional LERW and USTs as essential input. However, these bounds are not strong enough to plug into the bootstrap strategy presented at the end of Sect. 3.
